# Reconfiguring surface functions using visible-light-controlled metal-ligand coordination

**DOI:** 10.1038/s41467-018-06180-7

**Published:** 2018-09-21

**Authors:** Chaoming Xie, Wen Sun, Hao Lu, Annika Kretzschmann, Jiahui Liu, Manfred Wagner, Hans-Jürgen Butt, Xu Deng, Si Wu

**Affiliations:** 1 Institute of Fundamental and Frontier Sciences, University of Electronic Science and Technology of China, 610054 Chengdu, China; 2 Max Planck Institute for Polymer Research, 55128 Mainz, Germany; 3 Hefei National Laboratory for Physical Sciences at the Microscale, CAS Key Laboratory of Soft Matter Chemistry, Anhui Key Laboratory of Optoelectronic Science and Technology, Innovation Centre of Chemistry for Energy Materials, Department of Polymer Science and Engineering, University of Science and Technology of China, Hefei, 230026 China

## Abstract

Most surfaces are either static or switchable only between “on” and “off” states for a specific application. It is a challenge to develop reconfigurable surfaces that can adapt to rapidly changing environments or applications. Here, we demonstrate fabrication of surfaces that can be reconfigured for user-defined functions using visible-light-controlled Ru–thioether coordination chemistry. We modify substrates with Ru complex Ru-H_2_O. To endow a Ru-H_2_O-modified substrate with a certain function, a functional thioether ligand is immobilized on the substrate via Ru–thioether coordination. To change the surface function, the immobilized thioether ligand is cleaved from the substrate by visible-light-induced ligand dissociation, and then another thioether ligand with a distinct function is immobilized on the substrate. Different thioethers endow the surface with different functions. Based on this strategy, we rewrite surface patterns, manipulate protein adsorption, and control surface wettability. This strategy enables the fabrication of reconfigurable surfaces with customizable functions on demand.

## Abstract (web summary)

Configuring surfaces on-demand for desired functionalities is an ongoing challenge. Here, diverse and tailorable modifications of quartz and porous silica surfaces that are rapidly and reversibly switchable by the use of visible light are achieved via ruthenium-thioether coordination.

## Introduction

Manipulating surface properties is important for self-cleaning, guiding liquid flow, controlling protein adsorption, regulating cell adhesion, and many other applications^1–5^. The properties of stimuli-responsive surfaces can be changed using external stimuli, such as light, heat, electric field, CO_2_, glucose, and pH^6–12^. For example, the surface properties can be manipulated using pH-controlled dynamic imine-based covalent reaction^13,14^. Among these stimuli, light has attracted increasing attention because of its high spatiotemporal resolution and remote-control mechanism. To date, a variety of photolysis and photocoupling reactions have been utilized to control surface function^15–20^. However, most of the reported photoreactions result in only static and irreversible surface functions because of the irreversible formation or photocleavage of C–C or C–O bonds. Reversible surface functions have been realized by modifying surfaces with photoswitchable compounds such as azobenzene^21–26^, spiropyran^27–29^, dithienylethene^30^, and synthetic molecular shuttles^31^. Such surfaces can switch only between two functional states because photoswitchable compounds interconvert between two isomers under ultraviolet (UV)/visible-light irradiation. Reconfigurable surfaces, which can be converted into multiple states, have been constructed using photoreactions such as the photodynamic disulfide exchange reaction^18^, thiol–quinone methide reaction^32^, addition–fragmentation chain-transfer reaction^33^, and thiol–disulfide interconversion^19^. These photoreactions enable fabrication of customized surfaces on demand. However, all these reconfigurable surfaces are manipulated with UV light, which can damage biological components and shorten the lifetime of organic/polymeric materials. Furthermore, UV light cannot penetrate deeply into tissue, which is not suitable for manipulating biointerfaces in the body (e.g., surfaces on implants). Compared to UV light, visible light is not invasive, and red light in the visible region can penetrate deeply into tissue. Therefore, it is highly desirable to construct reconfigurable surfaces that are controllable by visible light.

Ligand photosubstitution is a powerful photoreaction for constructing reconfigurable surfaces. In ligand photosubstitution, a ligand in a metal complex is replaced by another one under light irradiation^34^. In particular, ligands on some Ru complexes can be substituted under visible light^35–37^. Light-controlled ligand substitution has been applied for uncaging neurotransmitters^38,39^, activating anticancer drugs^40–43^, controlling drug release^44,45^, actuating hydrogels^46,47^, and photopatterning^48^. In particular, we have demonstrated that red light can pass through tissue and induce photosubstitution in Ru complexes in vivo^41^. Although the abovementioned studies use only one-way photosubstitution, photosubstitution reactions of some Ru complexes are reversible at ambient or elevated temperatures^49^. For example, thioethers can substitute for the coordinated water molecules in Ru complexes in the dark via thermal substitution; water molecules can also substitute for the coordinated thioether ligands in Ru complexes under light irradiation^50,51^. Therefore, Ru complexes can interconvert between two states (i.e., the water-coordinated and thioether-coordinated states) in solution via reversible ligand photosubstitution. However, ligand photosubstitution has never been used to construct a system that can be reconfigured among multiple states.

**Fig. 1:**
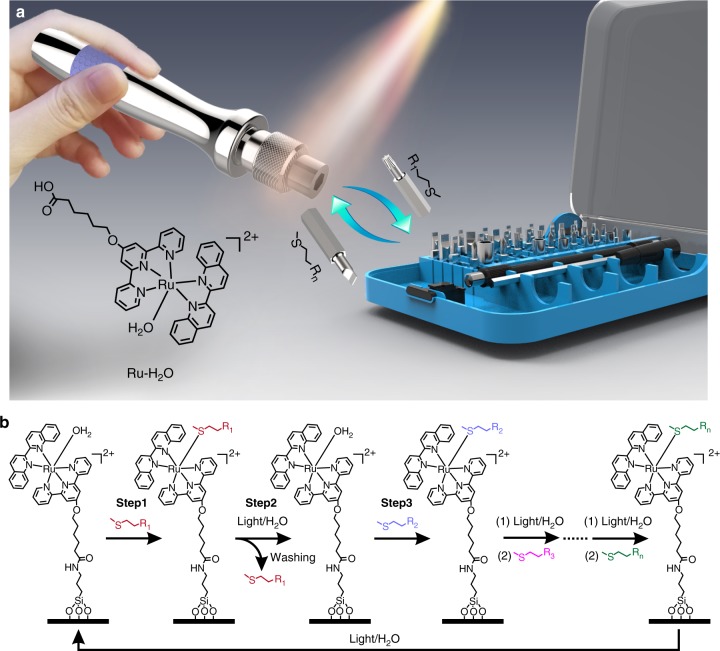
Concept and mechanism of reconfiguring surface functions using visible light. **a** Schematic diagram of the visible-light-controlled reconfigurable multi-functional platform. Ru-H_2_O acts as a screwdriver, which can take a functional bit (thioethers with different functional groups) and change the function via ligand substitution. **b** The mechanism of reconfiguring surfaces via ligand substitution. A thioether ligand substitutes the coordinated H_2_O molecule in the dark and a H_2_O molecule substitutes a thioether ligand under visible-light irradiation. This process can occur for many times to reconfigure the surface

In this work, we constructed a reconfigurable surface, which can be reconfigured into a number of functional states using visible-light-controlled metal–ligand coordination. In our design, the Ru complex [Ru(tpy-COOH)(biq)(H_2_O)](PF_6_)_2_ (hereafter denoted Ru-H_2_O, tpy-COOH = 6-2,2’:6’,2”-terpyridin-4’-yloxy hexanoic acid, biq = 2,2’-biquinoline) acts as the molecular “multi-bit screwdriver”, and the thioethers with different functional groups (MeSC_2_H_4_-R_1_, MeSC_2_H_4_-R_2_, MeSC_2_H_4_-R_3_…MeSC_2_H_4_-R_n_) act as the molecular bits (Fig. 1a). The removal of the bit on the screwdriver is driven by visible-light-induced photosubstitution, while the attachment of another bit to the screwdriver is automatically achieved in the dark via thermal substitution. To construct a reconfigurable surface, Ru-H_2_O is grafted onto a substrate (Fig. 1b). Substitution of the coordinated H_2_O molecule in Ru-H_2_O with the thioether (MeSC_2_H_4_-R_1_) endows the surface with the function of R_1_ (step 1 in Fig. 1b). To change the surface function to that of R_2_, MeSC_2_H_4_-R_1_ is first substituted by H_2_O under light irradiation (step 2). After washing with water and acetone to remove MeSC_2_H_4_-R_1_, the coordinated H_2_O is then substituted by MeSC_2_H_4_-R_2_ in the dark (step 3). The surface can be reconfigured into user-defined functions using different thioethers based on the approach in Fig. 1b. This approach enables fabrication of reconfigurable surfaces with customized functions. We demonstrate rewriting surface patterns, manipulating protein adsorption, and controlling wettability based on visible-light-controlled metal–ligand coordination.

## Results

### Ru–thioether coordination in aqueous solutions

Ru-H_2_O was designed for this study; it has a carboxylic group for surface modification and a coordinated H_2_O molecule that can be substituted by thioethers (Fig. 1). Ru-H_2_O was synthesized via a multi-step route and fully characterized using ^1^H nuclear magnetic resonance (NMR) spectroscopy, ^13^C NMR spectroscopy, H-H correlation spectroscopy (COSY) spectrum, and mass spectrometry (Supplementary Figs. 1–8).

**Fig. 2:**
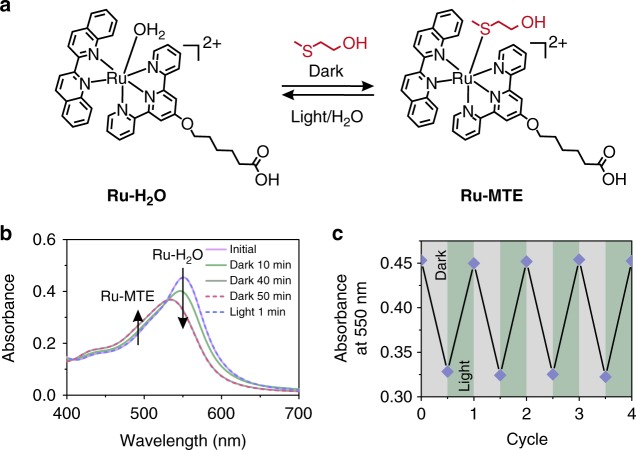
Visible-light-controlled Ru–thioether coordination in solution. **a** Interconversion of Ru-H_2_O and Ru-MTE in solution. **b** UV–vis absorption spectra of an aqueous solution containing Ru-H_2_O (1 mM) and MTE (10 mM) in the dark for 10, 40, and 50 min and subsequently irradiated with green light for 1 min. **c** Absorption changes in the aqueous solution containing Ru-H_2_O (1 mM) and MTE (10 mM) for alternating dark/light irradiation cycles

To demonstrate that the Ru–thioether bond is dynamic and reversible, we studied the coordination between Ru-H_2_O and a model thioether compound 2-(methylthio)ethanol (MTE) using UV–vis absorption spectroscopy (Fig. 2a). When Ru-H_2_O (1 mM) and MTE (10 mM) were mixed in water, the absorption band was located at 550 nm, which is attributed to the metal-to-ligand charge transfer band of Ru-H_2_O. The absorption band blueshifted to 535 nm and the absorbance decreased when the mixture was kept in the dark (Fig. 2b). This spectral change is identical to the observations of the formation of the Ru–thioether coordination bond reported in the literature^50,51^, which indicated MTE coordinated with the Ru center. The absorption band did not further blueshift after 40 min, indicating equilibrium was reached. Then, the sample was irradiated with green light (530 nm, 50 mW cm^–2^) for 1 min. The absorption band returned to the original position. This result showed that MTE was substituted by water upon irradiation. The existence of an isosbestic point at 530 nm suggested that a single reaction occurred. The photosubstitution of the Ru complex can also be induced by UV, blue, and red light in solution because the Ru complex has a broad absorption band in the UV and visible regions (Supplementary Fig. 9 and Supplementary Note 1).

To demonstrate the reversibility of the substitution, we studied the ligand substitution for four cycles using absorption spectroscopy (Fig. 2c). In each cycle, an aqueous mixture of Ru-H_2_O (1 mM) and MTE (10 mM) was kept in the dark for 40 min and then irradiated with green light (530 nm, 50 mW cm^–2^) for 1 min. The absorption change shows that the ligand substitution was fully reversible (Fig. 2c).

We also studied the ligand substitution using ^1^H NMR spectroscopy (Supplementary Fig. 10 and Supplementary Note 2). The ^1^H NMR data confirmed that MTE coordinated with the Ru center in the dark and was substituted by a water molecule upon green-light irradiation. The formation and cleavage of the Ru–thioether bond can be cycled for at least 10 times under dark/light irradiation conditions (Supplementary Fig. 11a). The MTE-coordinated Ru centers were approximately 80% in each cycle (Supplementary Fig. 11b and Supplementary Note 3). The equilibrium constant *K* of the MTE/Ru-H_2_O coordination reaction was 107 ± 4 M^–1^ at 298 K (Supplementary Fig. 12 and Supplementary Note 4). The pseudo first-order rate constant k′_1_ was 1 × 10^–3^ s^–1^ at 298 K (Supplementary Fig. 13 and Supplementary Note 5). The formation of the Ru-MTE complex in the dark was also quantified using UV–vis absorption spectroscopy (Supplementary Fig. 14 and Supplementary Note 6). Based on these results, we conclude that the Ru–thioether bond is dynamic, and a reversible ligand substitution occurs under mild conditions without the addition of any other reagents such as catalysts or photoinitiators.

### Ru–thioether coordination on surfaces

Encouraged by the reversible ligand substitution in solution, we studied visible-light-controlled Ru–thioether coordination on surfaces. To fabricate a reconfigurable surface, a quartz substrate was modified with (3-aminopropyl)triethoxysilane (APTES) and then Ru-H_2_O was grafted onto the substrate via amidation. Subsequently, we vertically inserted the Ru-H_2_O-modified quartz substrate (1 × 2 cm^2^) into an MTE aqueous solution (10 mM) in a quartz cuvette and studied the ligand substitution at the surface using absorption spectroscopy. The absorption band of the grafted Ru-H_2_O at 550 nm decreased and slightly blueshifted in the dark. The absorption band returned to the initial state after green-light irradiation (530 nm, 40 mW cm^–2^) for 10 min (Supplementary Fig. 15). The spectral change was similar to that observed in solution, which indicated that the ligand substitution on the surface was reversible.

We studied the released photoproduct from the surface using mass spectrometry, which showed that MTE was intact after photoinduced releasing from the surface (Supplementary Fig. 16 and Supplementary Note 7). Moreover, the surfaces were stable when stored in a fridge at –4 °C, in air and under vacuum for 5 days in the dark (Supplementary Fig. 17 and Supplementary Note 8). The photostability of the Ru-MTE-modified surfaces in solution (Supplementary Fig. 18 and Supplementary Note 9) and in air (Supplementary Fig. 19 and Supplementary Note 10) was also studied. The Ru-MTE-modified surfaces were stable in dry air even under UV or visible light irradiation.

The reversible substitution on the surface was quantified using X-ray photoelectron spectroscopy (XPS). The reversible change of S 2p signal in the XPS spectra revealed that MTE was grafted on the surface in the dark and cleaved from the surface after light irradiation (Supplementary Fig. 20 and Supplementary Note 11). The quantitative analysis of XPS spectra also showed that the substitution was reversible for at least 10 cycles (Supplementary Table 1). Almost all thioether ligands were cleaved from the surface after light irradiation and washing. In the dark, 49.6 to 70.9% of Ru centers (average 62%) on the surface was coordinated with MTE (Supplementary Table 1).

### Rewriting surface patterns with visible light

**Fig. 3:**
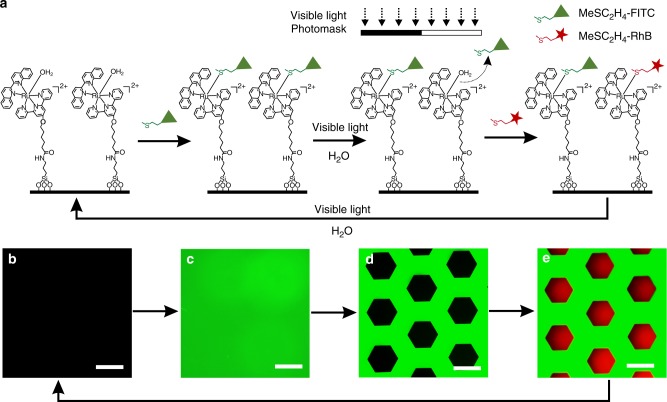
Rewriting surface patterns on the Ru-H_2_O-modified substrate using visible light. **a** Schematic illustration of the rewriting surface patterns. The fluorescence microscopy images of **b** the Ru-H_2_O-modified substrate, **c** Ru-MeSC_2_H_4_-FITC-modified substrate, **d** patterned substrate consisting of a Ru-MeSC_2_H_4_-FITC-modified part (green) and Ru-H_2_O-modified part (dark), and **e** patterned substrate consisting of a Ru-MeSC_2_H_4_-FITC-modified part (green) and Ru-MeSC_2_H_4_-RhB-modified part (red). Scale bars are 300 µm

To demonstrate the reconfigurable features of the Ru-H_2_O-modified surface, we synthesized thioether-containing fluorescein isothiocyanate (MeSC_2_H_4_-FITC) and thioether-containing rhodamine B isothiocyanate (MeSC_2_H_4_-RhB) to create rewritable patterns (Fig. 3a). First, the Ru-H_2_O-modified surface was immersed in an aqueous solution of MeSC_2_H_4_-FITC (10 mM) in the dark for 2 h and then washed with water and acetone. The non-fluorescent surface (Fig. 3b) developed a strong green fluorescence (Fig. 3c), showing MeSC_2_H_4_-FITC was successfully immobilized on the surface. Then, the substrate was wetted with water, covered by a photomask, and irradiated using green light (530 nm, 40 mW cm^–2^) for 10 min. The disappearance of the fluorescence in the exposed regions demonstrated that MeSC_2_H_4_-FITC was cleaved from the surface (Fig. 3d). After that, the substrate was immersed into an aqueous solution of MeSC_2_H_4_-RhB (10 mM) in the dark for 2 h and then washed with water and acetone. Red fluorescence appeared in the previously exposed regions (Fig. 3e), which suggested MeSC_2_H_4_-RhB was immobilized on the surface. Moreover, the well-defined structure showed that the visible-light-induced ligand substitution can pattern different functional ligands with a good spatial resolution. Importantly, the patterned surface returned to the original state upon irradiation with green light in water, revealing the rewritable feature of the Ru-H_2_O-modified surface.

### Manipulating protein adsorption with visible light

**Fig. 4:**
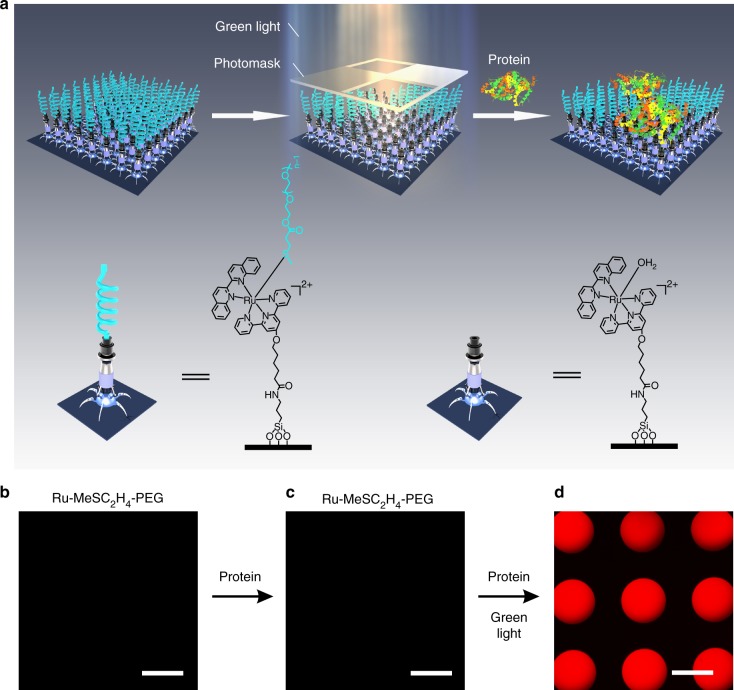
Controlling protein adsorption with visible light. **a** Schematic illustration of the conversion of a protein-resistant surface into a protein-adsorptive surface with light. The fluorescent images of the **b** Ru-MeSC_2_H_4_-PEG-modified surface, **c** Ru-MeSC_2_H_4_-PEG-modified surface after immersion in a solution of fluorescently labeled BSA (0.5 mg mL^–1^) for 2 h and washing with an aqueous solution of NaCl (1 mM, pH = 9), and **d** protein pattern fabricated using the approach in **a**. Scale bars are 300 µm

Surfaces that either resist or enhance protein adsorption are important for biomedical applications. Visible-light-controlled metal–ligand coordination enables reconfiguration of a surface from a protein-repellent state to a protein-adsorptive state. To fabricate a protein-resistant surface, a thioether-terminated polyethylene glycol ligand (MeSC_2_H_4_-PEG) was synthesized, which can form a dynamic coordination bond with Ru-H_2_O (Supplementary Fig. 21). The coordination of MeSC_2_H_4_-PEG and Ru-H_2_O was reversible in solution (Supplementary Fig. 22). To fabricate a protein-resistant surface, MeSC_2_H_4_-PEG was immobilized on a Ru-H_2_O-modified surface via Ru–thioether coordination (Fig. 4a, left). The Ru-MeSC_2_H_4_-PEG-modified surface was non-fluorescent (Fig. 4b). Then, the Ru-MeSC_2_H_4_-PEG-modified surface was immersed into a solution of fluorescently labeled bovine serum albumin (BSA) (0.5 mg mL^–1^) for 2 h and washed with an aqueous solution of NaCl (1 mM, pH = 9). After this treatment, the Ru-MeSC_2_H_4_-PEG-modified surface was still non-fluorescent (Fig. 4c), showing the surface was resistant to protein adsorption. To convert the protein-resistant surface into a protein-adsorptive surface, the surface was wetted with the fluorescently labeled BSA solution and irradiated with masked green light (530 nm, 40 mW cm^–2^) for 10 min (Fig. 4a, middle and right). The MeSC_2_H_4_-PEG on the exposed areas was cleaved from the surface and the fluorescently labeled BSA was captured by the exposed regions via electrostatic interactions (Fig. 4d). These results demonstrate that visible-light-controlled metal–ligand coordination can manipulate protein adsorption.

**Fig. 5:**
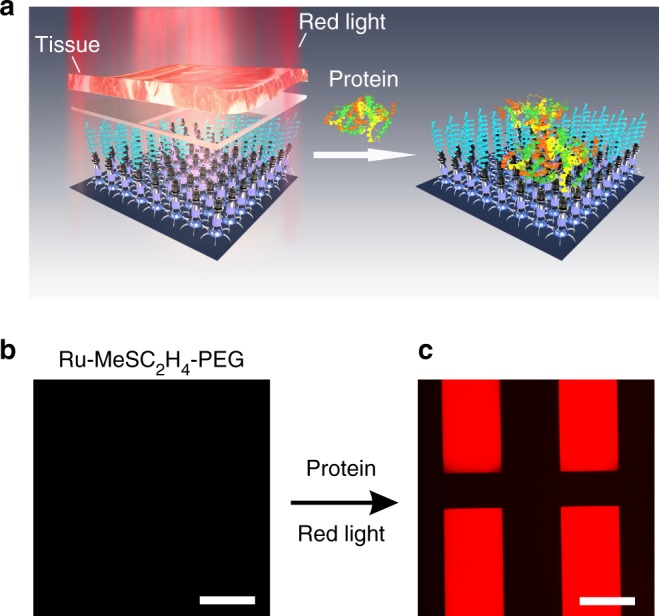
Protein resistance and capture under tissue. **a** Schematic illustration of the fabrication of a protein pattern using red light after the light passes through a piece of tissue. Florescence microscopy images of a Ru-MeSC_2_H_4_-PEG-modified surface wetted with the fluorescently labeled BSA solution (**b**) before and **c** after red-light irradiation through the tissue and photomask. Scale bars are 300 µm

Manipulating protein adsorption on biomaterials in the body (e.g., implants) with light requires light that can penetrate deeply into tissue. We have demonstrated that red light can pass through biological tissue and activate Ru complexes with absorption and photoreactivity properties similar to Ru-MeSC_2_H_4_-PEG^41,52^. To test the potential deep-tissue applications of the Ru-MeSC_2_H_4_-PEG-modified surface, we placed a piece of 4-mm-thick pork tissue between the Ru-MeSC_2_H_4_-PEG-modified surface and a red laser (671 nm, 110 mW cm^–2^). The surface was wetted with the fluorescently labeled BSA solution (0.5 mg mL^–1^) and irradiated with masked red light for 40 min (Fig. 5a). The exposed areas changed from non-fluorescent to fluorescent after light illumination, which indicated the proteins were adsorbed on the exposed areas (Fig. 5b, c). The laser wavelength (671 nm) is in the therapeutic window (600–1000 nm) and the laser intensity (110 mW cm^–2^) is lower than the maximum permissible exposure for skin exposure (200 mW cm^–2^)^53,54^. Therefore, manipulating protein adsorption using our system is a noninvasive method for deep-tissue applications. Furthermore, we tested biocompatibility of Ru-H_2_O-modified surfaces by measuring cell viability with a quartz substrate or a Ru-H_2_O-modified quartz substrate (Supplementary Fig. 23 and Supplementary Note 12). The biocompatibility of Ru-H_2_O-modified quartz is comparable to unmodified quartz.

### Controlling wettability with visible light

**Fig. 6:**
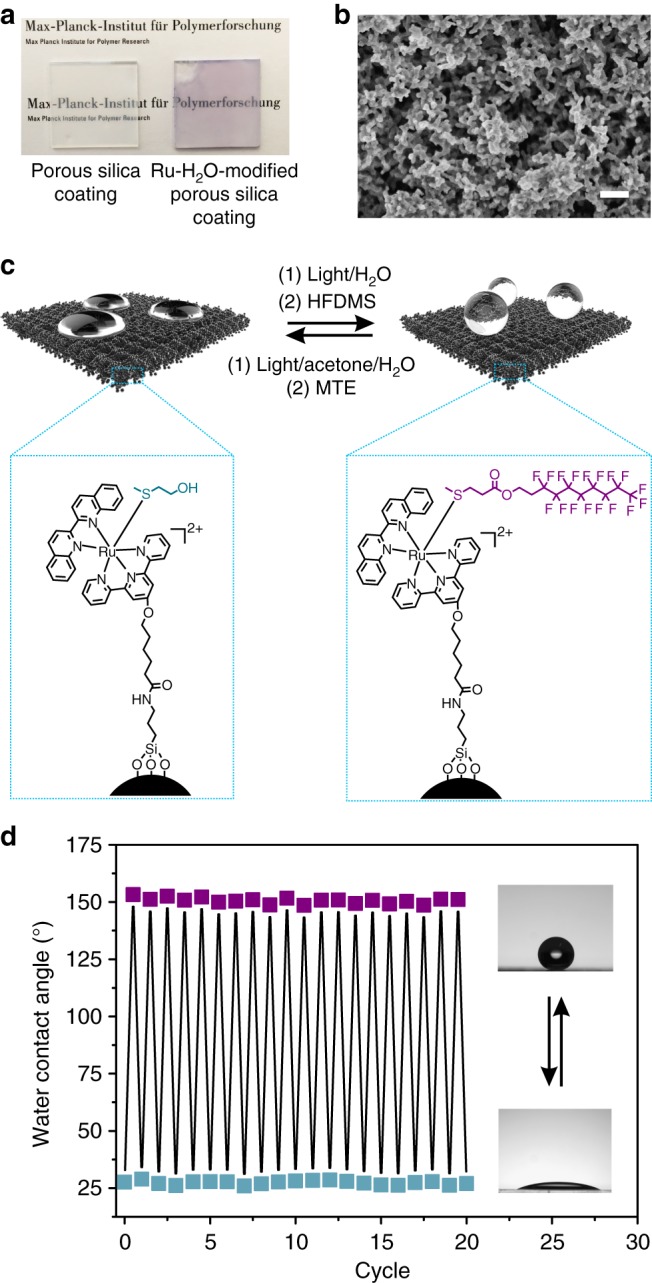
Visible-light-controlled reversible switching of the surface wettability. **a** Photographs of the porous silica coating before and after modification with Ru-H_2_O. **b** Scanning electron microscope (SEM) image of the Ru-H_2_O-modified porous silica coating. Scale bar is 400 nm. **c** Schematic illustration of the reversible hydrophilic-to-superhydrophobic transitions based on visible-light-controlled metal–ligand coordination. **d** Change in the static contact angles when the ligands on the surface were interconverted between MTE and HFDMS. Blue squares indicate MTE ligands on the surface. Purple squares indicate HFDMS ligands on the surface

Another application of visible-light-controlled metal–ligand coordination is manipulating the wettability of surfaces. We can adjust the wettability using suitable thioether ligands and switch the wettability with visible light. As a proof of concept, we prepared a surface that showed reversible hydrophilic-to-superhydrophobic transitions (Fig. 6). For this purpose, we prepared a porous silica coating, which was created from a candle soot template developed in our previous work^55^ (Fig. 6a, b and Supplementary Fig. 24). The coating was grafted with APTES and subsequently modified with Ru-H_2_O. The coating was purple after it was modified with Ru-H_2_O (Fig. 6a, right).

MTE was used as the hydrophilic thioether ligand and (3,3,4,4,5,5,6,6,7,7,8,8,9,9,10,10,10-heptadecafluorodecyl)(methyl)sulfane (HFDMS) was used as the hydrophobic thioether ligand (Supplementary Fig. 25). The coordination of HFDMS and Ru-H_2_O was also reversible in solution (Supplementary Fig. 26). The coating with these two thioether ligands can be interconverted via visible-light-controlled metal–ligand coordination (Fig. 6c). First, the Ru-MTE-modified coating was prepared by immersing the Ru-H_2_O-modified substrate into an aqueous solution of MTE (10 mM) for 2 h in the dark, and the coating had a static water contact angle of 27 ± 2° (Fig. 6d). The low contact angle is because of the nanostructure of the coating and the hydrophilic feature of the surface groups. Subsequently, MTE was cleaved from the surface by irradiating the coating with green-light irradiation (530 nm, 40 mW cm^–2^, 10 min) in water. After that, HFDMS was immobilized on the substrate by immersing the substrate into an acetone/H_2_O (1/1) solution of HFDMS (10 mM) in the dark for 2 h and washing with acetone. After drying, the coating had a static water contact angle of 154 ± 2° and a roll-off angle less than 2°. This result shows the coating changed from hydrophilic to superhydrophobic because of the low surface free energy of the fluorocarbon chains. To switch the coating back to the hydrophilic state, the Ru-HFDMS-modified coating was irradiated with green light (530 nm, 40 mW cm^–2^, 10 min) in an acetone/H_2_O mixture and immersed into an aqueous solution of MTE (10 mM) for 2 h in the dark. The hydrophilic-to-superhydrophobic transitions were recyclable (Fig. 6d). Our reconfigurable surfaces based on the Ru–thioether dynamic bond are different from conventional photoswitchable surfaces based on photoisomerization. Conventional photoswitchable surfaces have two steady states. We can endow the surface with multiple steady states. As a proof of concept, we demonstrated the wettability of the surface can also be switched between a hydrophilic state and a hydrophobic state using another thioether (Supplementary Fig. 27 and Supplementary Note 13).

## Discussion

In conclusion, we showed a universal strategy for constructing dynamic surfaces that can be reconfigured into user-defined functional states using visible-light-controlled metal–ligand coordination. As a proof of concept, we demonstrated rewriting surface patterns using Ru-MeSC_2_H_4_-FITC and Ru-MeSC_2_H_4_-RhB coordination, manipulating protein adsorption using Ru-MeSC_2_H_4_-PEG coordination, and controlling wettability using Ru-MTE, Ru-HFDMS and Ru-DMS coordination. In principle, customizable surfaces can be readily obtained using desirable thioether ligands, which are either commercially available or can be easily synthesized by modifying MTE, 3-(methylthio)propionic acid, or 2-(methylthio)ethylamine. The highly customizable functions of thioether ligands can endow the Ru–thioether-modified surfaces with different applications. The functions and applications can be easily reconfigured with visible light, which is a noninvasive stimulus. Importantly, the reported reconfigurable surfaces were also responsive to red light, which can penetrate deeply into tissue for biomedical applications. We believe that our strategy is versatile for customizable surface functionalization and opens exciting opportunities for a wide range of applications.

## Methods

### Synthesis

Detailed procedures for the synthesis and characterization of the Ru complexes and ligands are provided in the Supplementary Information.

### Modifying quartz substrates with Ru-H_2_O

First, a quartz substrate (2 × 2 cm^2^) was immersed in piranha solution (H_2_SO_4_/H_2_O_2_ = 2/1) for 1 h at 90°, washed with water, ethanol, and acetone, and dried using a stream of N_2_. Then, the substrate was immersed in an ethanol solution of 1% APTES for 24 h, washed with water, ethanol, and acetone, and dried using a stream of N_2_. Afterwards, the substrate was immersed into a dry dichloromethane (DCM) solution (10 mL) of Ru-H_2_O (100 mg, 0.097 mmol), *N*-(3-dimethylaminopropyl)-*N*’-ethylcarbodiimide hydrochloride (EDC, 68 mg, 0.355 mmol), and 4-(dimethylamino)pyridine (DMAP, 40 mg, 0.327 mmol) for 24 h before it was washed with water, ethanol, and acetone, and finally dried using a stream of N_2_.

### Rewriting surface patterns with visible light

First, FITC and RhB were separately dissolved in dimethyl sulfoxide (20 mg mL^–1^). Subsequently, the FITC (0.96 mL, 0.05 mmol) and RhB (1.35 mL, 0.05 mmol) solutions were added into 4.04 mL and 3.65 mL aqueous MTE solutions (0.05 mmol), respectively, and stirred overnight to obtain FITC- and RhB-modified thioethers (MeSC_2_H_4_-FITC and MeSC_2_H_4_-RhB). Afterwards, the Ru-H_2_O-modified substrate was immersed into a MeSC_2_H_4_-FITC solution (10 mM) in the dark for 2 h, and washed with water and acetone. After drying with a stream of N_2_, the Ru-MeSC_2_H_4_-FITC-modified substrate was wetted by water, covered by a photomask, and irradiated using green light (530 nm, 40 mW cm^–2^) for 10 min. After it was washed and dried again, the substrate was immersed into a MeSC_2_H_4_-RhB solution (10 mM) in the dark for 2 h to form a patterned surface. To regenerate the Ru-H_2_O-modified surface, the patterned surface was irradiated with green light (530 nm, 40 mW cm^–2^) in water for 10 min. The substrate was imaged using an inverted fluorescence microscope (DMi8, Leica).

### Manipulating protein adsorption with visible light

A Ru-H_2_O-modified substrate was immersed into an aqueous solution of MeSC_2_H_4_-PEG (10 mL, 10 mM) in the dark overnight, washed with water and acetone, and dried using a stream of N_2_. Afterwards, the substrate was wetted by the fluorescently labeled BSA (0.5 mg mL^–1^) solution and irradiated with green light (530 nm, 40 mW cm^–2^) with a photomask for 10 min. Subsequently, the light was turned off, and the substrate was kept in the dark for 10 min. After washing and drying, the substrate was imaged using an inverted fluorescence microscope.

For the deep-tissue protein adsorption experiment, a piece of 4-mm-thick pork tissue and a red laser (671 nm, 110 mW cm^–2^) were used. First, the Ru-H_2_O-modified substrate was immersed into an aqueous solution of MeSC_2_H_4_-PEG (10 mL, 10 mM) in the dark overnight. Then, the substrate was washed with water and acetone, and dried using a stream of N_2_. Afterwards, the substrate was wetted by the fluorescently labeled BSA solution (0.5 mg mL^–1^) and placed between the red laser and a photomask covered with the tissue. After irradiation for 40 min, the laser was turned off, and the substrate was kept in the dark for 10 min. After washing and drying, the substrate was imaged using an inverted fluorescence microscope.

### Ru-H_2_O-modified porous silica coating

The porous silica coating (2 × 2 cm^2^) was prepared according to our previous work^55^. First, the candle soot-coated substrate was deposited by tetraethoxysilane using chemical vapor deposition for 72 h. Afterwards, the substrate was heated at 600 °C in air for 4 h to remove the carbon cores. Subsequently, the substrate was treated with oxygen plasma for 2 s and then placed into an ethanol solution of 1% APTES for 24 h. After that, the substrate was immersed in a dry DCM solution (10 mL) of Ru-H_2_O (100 mg, 0.097 mmol), EDC (68 mg, 0.355 mmol), and DMAP (40 mg, 0.327 mmol) for 24 h. After washing and drying, the Ru-H_2_O-modified porous silica coating was obtained.

### Reversible surface wettability

To obtain a hydrophilic surface, the Ru-H_2_O-modified porous silica coating was immersed in an aqueous solution of MTE (10 mL, 10 mM) for 2 h in the dark, washed with acetone and water, and dried using a stream of N_2_. To switch the hydrophilic surface to a superhydrophobic/hydrophobic surface, the hydrophilic Ru-MTE-modified coating was immersed into water and irradiated with green light (530 nm, 40 mW cm^–2^) for 10 min to cleave the MTE, washed with acetone and water, and dried using a stream of N_2_. After that, the coating was immersed into an acetone/H_2_O mixed solution of HFDMS or DMS (10 mL, 10 mM) for 2 h in the dark, washed with acetone, and dried with a stream of N_2_ to obtain a superhydrophobic/hydrophobic surface. The reversible hydrophilic-to-superhydrophobic/hydrophobic transitions were induced using the procedure mentioned above. The static water contact angles were measured using a contact angle meter (Dataphysics OCA35) by placing a 10 µL water droplet on each sample.

## Electronic supplementary material

Supplementary Information

## Data Availability

The data that support the findings of this study are available from the corresponding authors upon reasonable request.
