# Supplementary material for: Reconfiguring surface functions using visible-light-controlled metal-ligand coordination
Source: Nat Commun. 2018 Sep 21;9:3842. doi: 10.1038/s41467-018-06180-7 (PMC6154962; doi:10.1038/s41467-018-06180-7)
Supplement: Supplementary file 1 — Supplementary Information [file 41467_2018_6180_MOESM1_ESM.pdf]

Supplementary Information

Reconfiguring Surface Functions Using Visible-Light-Controlled Metal-Ligand Coordination

Xie et al.

Supplementary Methods

Materials. 2-(Methylthio)ethanol (99%), 2,2'-biquinoline (biq, ⩾ 99%), N,N'-dicyclohexylcarbodiimide (DCC, ⩾99%), 4-(dimethylamino)pyridine (DMAP, ⩾98%), N-(3-dimethylaminopropyl)-N'-ethylcarbodiimide hydrochloride (EDC, ⩾ 99%), Rhodamine B isothiocyanate (RhB), and fluorescein isothiocyanate (FITC) were purchased from Sigma-Aldrich. $RuCl_3$•$3H_2O$ (99.9%), ε-caprolactone (99%) and 1H,1H,2H,2H-perfluoro-1-decanol (97%) were purchased from Alfa Aesar. Silver hexafluorophosphate ($AgPF_6$, 98%), potassium hexafluorophosphate (KPF_6, 98%), and tetraethoxysilane (98%) were purchased from Acros Organics. 3-(Methylthio)propionic acid (>98%) and polyethylene glycol monomethyl ether 2000 (PEG2000) were purchased from Tokyo Chemical Industry. Dodecyl methyl sulfide was purchased from Santa Cruz. The fluorescently labeled bovine serum albumin (BSA, Alexa Fluor® 680 conjugate) was purchased from Life Technologies. All chemicals were used without further purification.

Characterization. NMR spectra were measured using Bruker Spectrospin NMR spectrometers (250 MHz, 300 MHz, and 500 MHz). UV-vis absorption spectra were measured using a Lambda 900 spectrometer (Perkin Elmer). The molecular weights were determined using mass spectrometry (Bruker Time-of-flight MS Reflex III). The morphologies of the Ru-H_2O-modified porous coatings were measured using a scanning electron microscope (SEM; LEO Gemini 1530). The water contact angle was measured using a contact angle meter (Dataphysics OCA35). The fluorescence microscopy images of the patterns were observed using an inverted fluorescence microscope (DMi8, Leica). The surface elements analysis was performed using an X-ray photoelectron spectrometer (XPS; Kratos, Manchester, England).

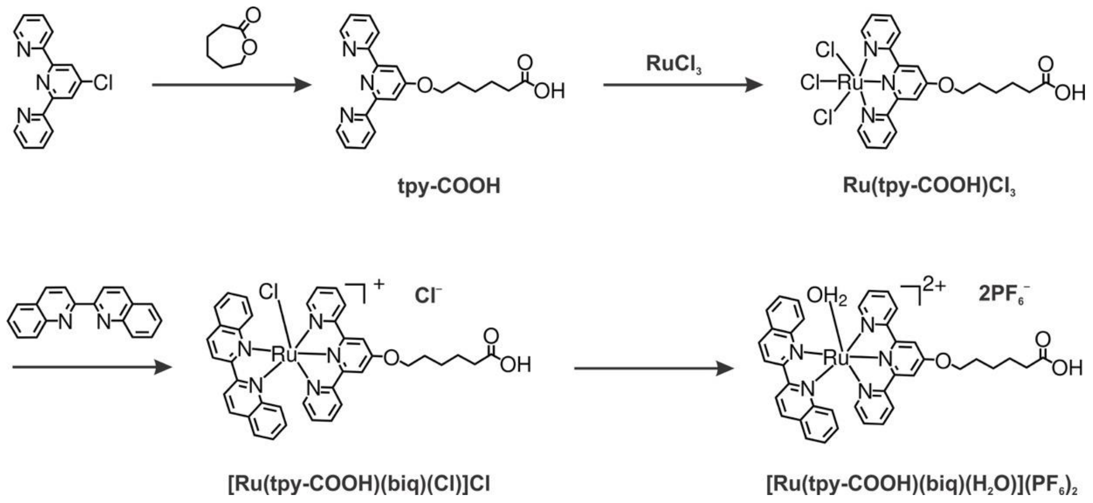

Supplementary Figure 1. Route for the synthesis of [Ru(typ-COOH)(biq)(H_2O)](PF_6)$_2$ (Ru-H_2O).

Synthesis of 6-(2,2':6',2''-terpyridin-4'-yloxy) hexanoic acid (tpy-COOH): It was synthesized according to the literature[1]. ^{1}H NMR (250 MHz, C_2D_6SO): δ 12.02 (s, 1H), 8.72 (d, *J* = 5.0 Hz, 2H), 8.62 (d, *J* = 7.5 Hz, 2H), 8.05 – 7.96 (m, 4H), 7.54 – 7.47 (m, 2H), 4.25 (t, *J* = 5.0 Hz, 2H), 2.26 (t, *J* = 5.0 Hz, 2H), 1.88 – 1.75 (m, 2H), 1.67 – 1.42 (m, 4H). ^{13}C NMR (75 MHz, C_2D_6SO): δ 174.32 (10a), 166.60 (5b), 156.57 (5a), 154.85 (4a), 149.05 (1), 137.30 (3), 124.50 (2), 120.86 (4), 106.75 (5), 67.82 (6), 33.63 (10), 28.03 (7), 24.86 (8), 24.20 (9). For atom notations, see Supplementary Figure 2, 3. ESI-MS (m/z): [M – H]$^+$, calcd. for $C_{21}H_{21}N_3O_3$, 363.16, found, 364.16.

Synthesis of Ru(tpy-COOH)Cl$_3$: $RuCl_3$•$3H_2O$ (220 mg, 0.84 mmol) and tpy-COOH (276.6 mg, 0.76 mmol) were combined in absolute ethanol (80 mL). The mixture was heated at reflux for 4 h.

After cooling to room temperature, the mixture was filtered. The filtered brown powder was washed with ethanol and diethyl ether sequentially to obtain the product (300 mg, 69%).

Synthesis of [Ru(tpy-COOH)(biq)(Cl)]Cl: Ru(tpy-COOH)Cl_3 (223 mg, 0.39 mmol) and 2,2'-biquinoline (biq, 100 mg, 0.39 mmol) were mixed in a 1:1 ethanol/H_2O mixture (20 mL). The mixture was bubbled with argon for 7 min before it was heated under reflux for 7 h under argon. Afterwards, the mixture was filtered while hot. The product was collected by evaporation under reduced pressure, and purified by column chromatography with silica gel (eluent: MeOH/DCM = 1:15 to 1:5) (132 mg, 43%). ^{1}H NMR (300 MHz, MeOD): δ 9.64 (d, *J* = 9.0 Hz, 1H), 8.95 (d, *J* = 9.0 Hz, 1H), 8.86 (d, *J* = 9.0 Hz, 1H), 8.65 (d, *J* = 9.0 Hz, 1H), 8.48 (d, *J* = 9.0 Hz, 2H), 8.30 – 8.20 (m, 4H), 9.93 – 7.78 (m, 7H), 7.46 (t, *J* = 6.0 Hz, 1H), 7.30 – 7.23 (m, 4H), 6.88 (d, *J* = 9.0 Hz, 1H), 4.47 (t, *J* = 6.0 Hz, 2H), 2.40 (t, *J* = 6.0 Hz, 2H), 2.06 – 1.95 (m, 2H), 1.85 – 1.60 (m, 4H). ^{13}C NMR (75 MHz, MeOD): δ 168.15 (10a), 163.49 (5b), 160.89 + 160.56 (T4a + T5a), 160.31 (A6a), 154.05 (T1 + B6a), 153.42 + 152.76 (A1a + B1a), 139.27 (A1), 138.73 (T3), 137.37 (B5), 132.01 (A6), 131.71 (B3 + B4), 130.61 (B4a), 130.45 + 130.36 (A2 + A3), 129.95 (A4), 129.75 (A4a), 129.62 (B2), 128.22 (T4), 125.17 +125.09 (B1 + T5), 121.83 (A5 + B6), 111.56 (T2), 71.22 (6), 35.14 (10), 29.78 (7), 26.73 (8), 25.90 (9). Also see Supplementary Figure 4, 5. MALDI-TOF (m/z): $[M]^+$, calcd. for $C_{39}H_{33}ClN_5O_3Ru$, 756.25, found, 755.95.

Synthesis of [Ru(tpy-COOH)(biq)(H_2O)](PF_6)$_2$: [Ru(typ-COOH)(biq)(Cl)]Cl (32 mg, 0.04 mmol) and $AgPF_6$ (36 mg, 0.14 mmol) were mixed in 4:1 acetone/H_2O mixture (5 mL). The mixture was bubbled with argon for 7 min and heated at reflux for 12 h under argon. After that, the mixture was filtered and concentrated to 1 mL under reduced pressure. The dark purple product

was precipitated from a saturated KPF_6 solution (20 mg, 49%). ^{1}H NMR (300 MHz, MeOD): δ 8.98 (m, 2H), 8.77 (d, *J* = 9.0 Hz, 1H), 8.69 (d, *J* = 9.0 Hz, 1H), 8.58 (d, *J* = 6.0 Hz, 2H), 8.44 – 8.37 (m, 3H), 8.25 (d, *J* = 6.0 Hz, 1H), 8.05 – 7.93 (m, 4H), 7.89 (d, *J* = 9.0 Hz, 1H), 7.48 (t, *J* = 9.0 Hz, 1H), 7.42 – 7.32 (m, 3H), 6.95 (d, *J* = 9.0 Hz, 1H), 4.57 (t, *J* = 6.0 Hz, 2H), 2.41 (t, *J* = 9.0 Hz, 2H), 2.10 – 1.98 (m, 2H), 1.86 – 1.64 (m, 4H). ^{13}C NMR (75 MHz, MeOD): δ 177.65 (10a), 169.57 (T5b), 163.71 (A6a), 160.98 (T1 + T5a), 160.38 (B6a), 155.02 (T4a), 153.95 (A1a), 152.30 (B1a), 140.27 (T3), 140.06 (A5), 138.33 (B5), 133.48 (A3), 132.46 (B3), 131.20 (A4), 130.90 (A4a), 130.68 + 130.63 (A2 + B4), 129.91 (B2), 129.49 (B4a), 129.42 (T4), 127.59 (A1), 125.87 (T5), 124.93 (B1), 122.41 (A6), 122.00 (B6), 112.52 (T2), 71.46 (6), 34.84 (10), 29.70 (7), 26.58 (8), 25.76 (9). For atom notations, see Supplementary Figure 6-8. MALDI-TOF (m/z): $[M - 2PF_6 - H]^+$, calcd. for $C_{39}H_{35}N_5O_4Ru$, 739.17, found, 740.14.

Synthesis of $MeSC_2H_4$-PEG: 3-(Methylthio)propionic acid (360 mg, 3 mmol), PEG2000 (2 g, 1 mmol) and DMAP (12 mg, 0.1 mmol) were mixed in dry DCM (40 mL). DCC (619 mg, 3 mmol) was dissolved in dry DCM (30 mL) and was slowly dropped into the mixture at 0 °C. Afterwards, the mixture was stirred for 2 days at room temperature. After that, the mixture was filtered, and concentrated to 5 mL under reduced pressure. The product was precipitated from cold diethyl ether (1.57 g, 75%). ^{1}H NMR (300 MHz, $CDCl_3$): δ 4.28 (t, *J* = 3.0 Hz, 2H), 3.93 – 3.54 (m, 183H), 3.39 (s, 3H), 2.79 (t, *J* = 9.0 Hz, 2H), 2.67 (t, *J* = 9.0 Hz, 2H), 2.14 (s, 3H). Also see Supplementary Figure 21.

Synthesis of HFDMS: 3-(Methylthio)propionic acid (500 mg, 4.2 mmol), 1H,1H,2H,2H-perfluoro-1-decanol (2 g, 4.3 mmol) and DMAP (52 mg, 0.43 mmol) were mixed in dry DCM (30

mL). DCC (900 mg, 4.3 mmol) was dissolve in dry DCM (20 mL), and was slowly dropped into the mixture at 0 °C. Afterwards, the mixture was stirred for 2 days at room temperature. The crude product was collected by evaporation under reduced pressure and purified by column chromatography with silica gel (eluent: ethyl acetate/hexane = 1:20) to yield 1.5 g (59%) of HFDMS as a yellow oil. ^{1}H NMR (300 MHz, $CDCl_3$): δ 4.41 (t, *J* = 6.0 Hz, 2H), 2.77 (t, *J* = 6.0 Hz, 2H), 2.64 (t, *J* = 6.0 Hz, 2H), 2.48 (m, 2H), 2.12 (s, 3H). Also see Supplementary Figure 25.

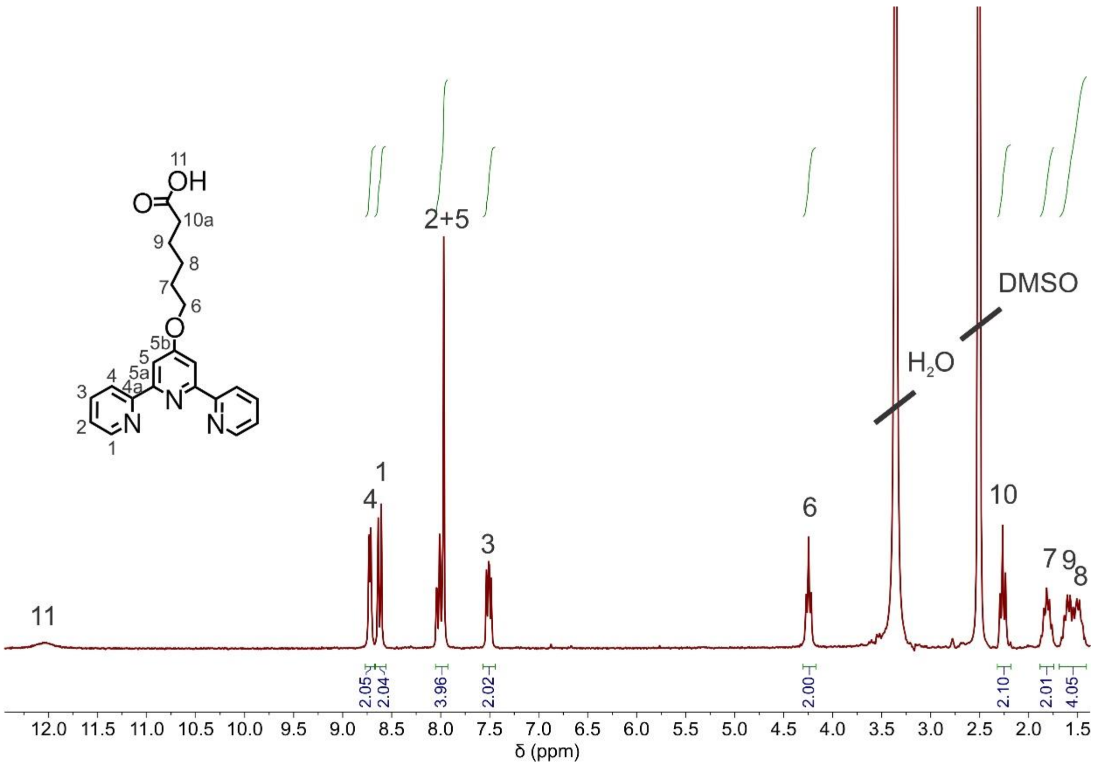

Supplementary Figure 2. ^{1}H NMR spectrum of tpy-COOH (250 MHz, DMSO-d_6).

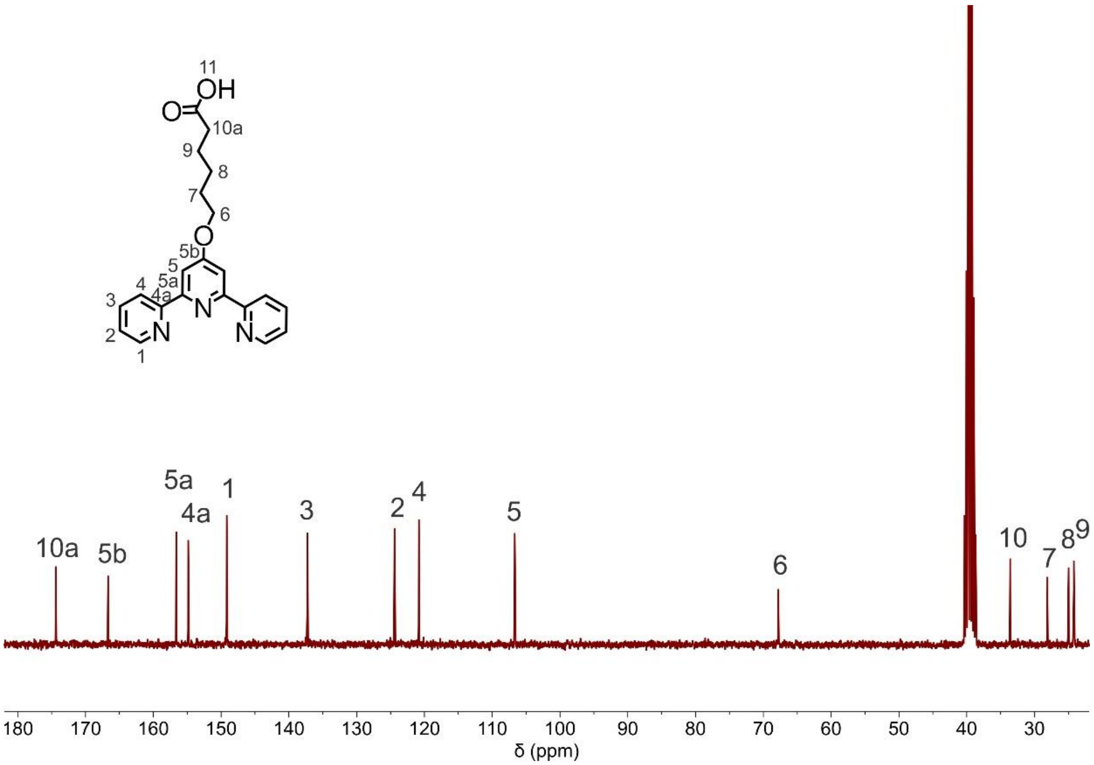

Supplementary Figure 3. ^{13}C NMR spectrum of tpy-COOH (75 MHz, DMSO-d_6).

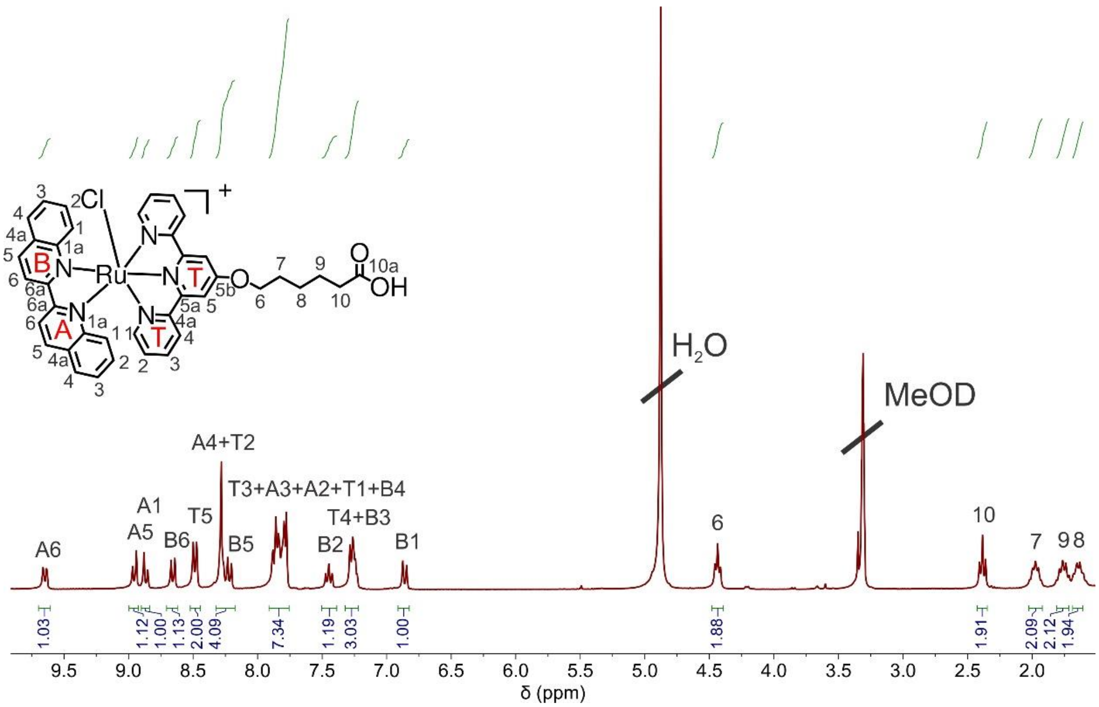

Supplementary Figure 4. ^{1}H NMR spectrum of [Ru(tpy-COOH)(biq)(Cl)]Cl (300 MHz, MeOD).

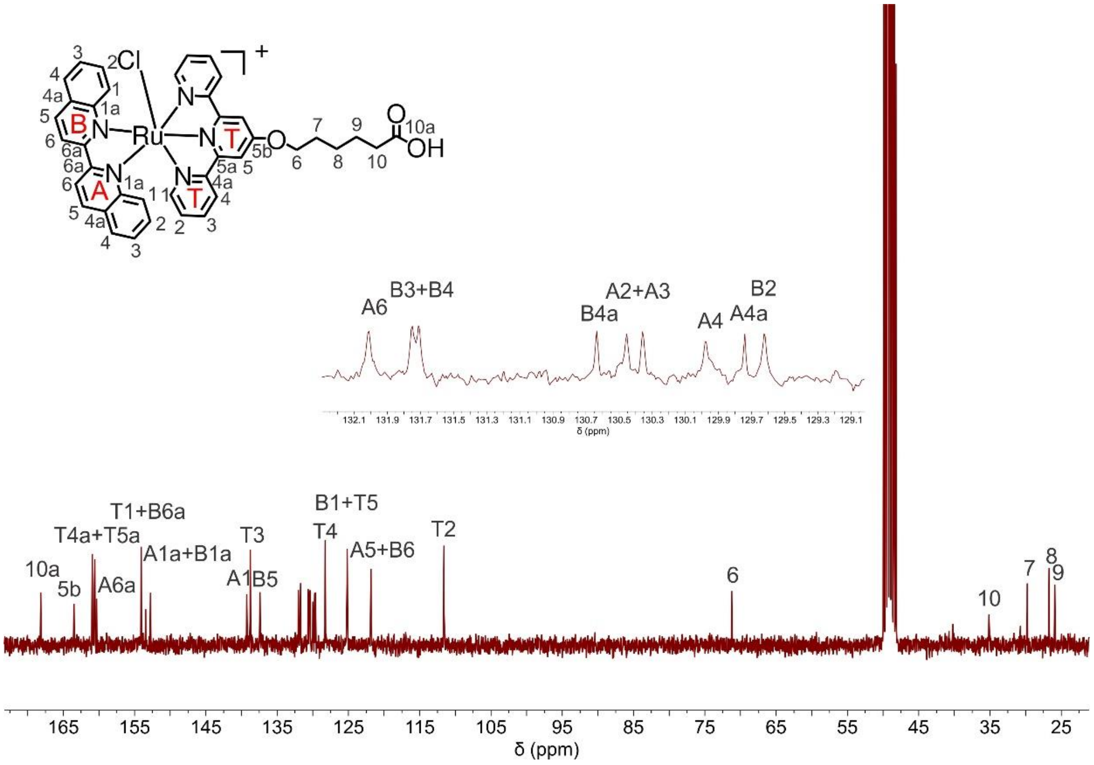

Supplementary Figure 5. ^{13}C NMR spectrum of [Ru(tpy-COOH)(biq)(Cl)]Cl (75 MHz, MeOD).

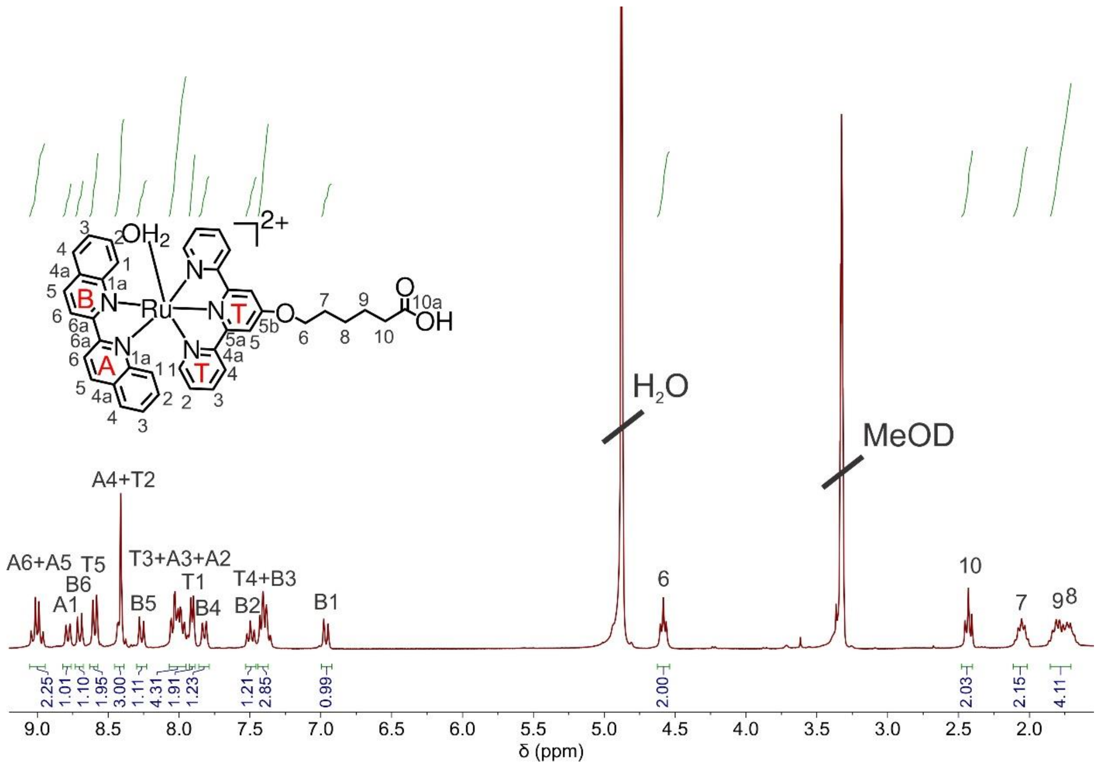

Supplementary Figure 6. ^{1}H NMR spectrum of [Ru(tpy-COOH)(biq)(H_2O)](PF_6)$_2$ (Ru-H_2O) (300 MHz, MeOD).

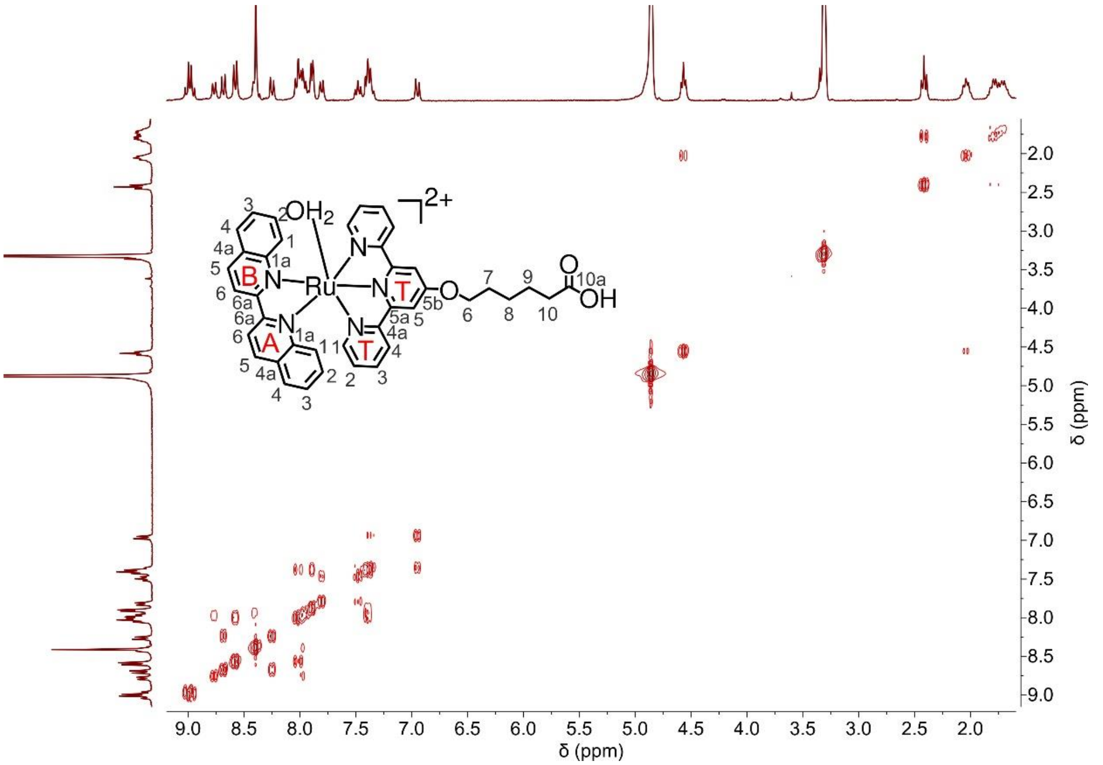

Supplementary Figure 7. H-H COSY spectrum of [Ru(tpy-COOH)(biq)(H_2O)](PF_6)$_2$ (300 MHz, MeOD).

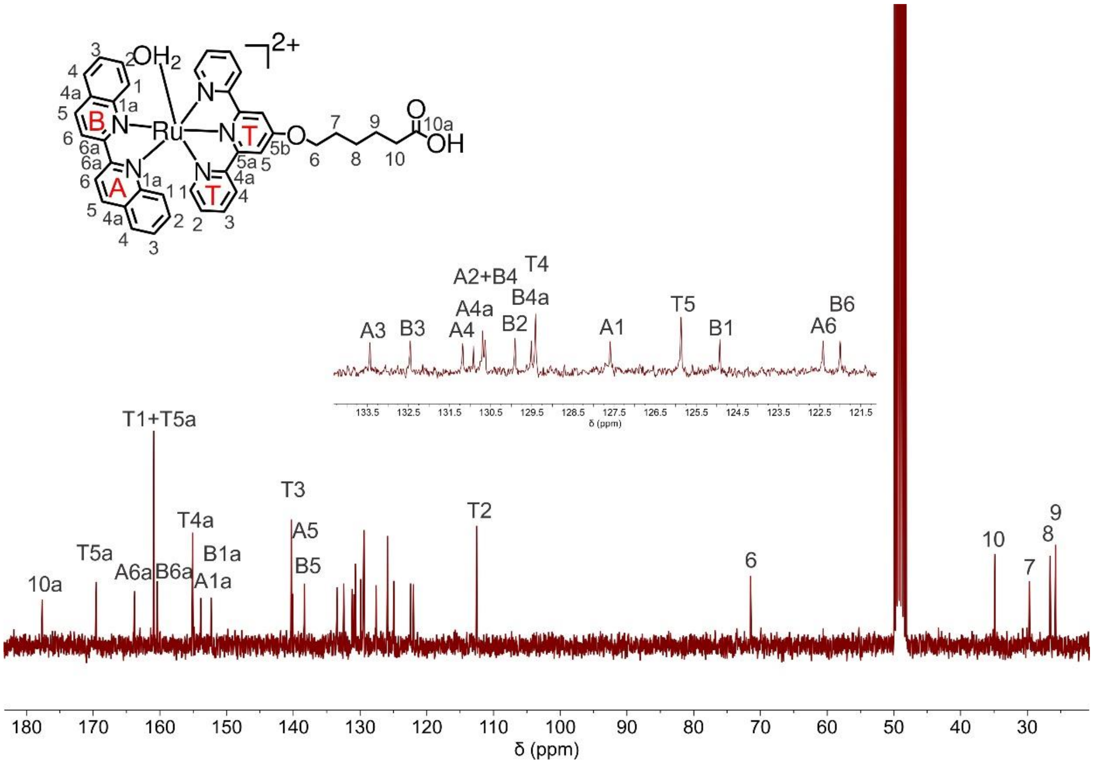

Supplementary Figure 8. ^{13}C NMR spectrum of [Ru(tpy-COOH)(biq)(H_2O)](PF_6)$_2$ (75 MHz, MeOD).

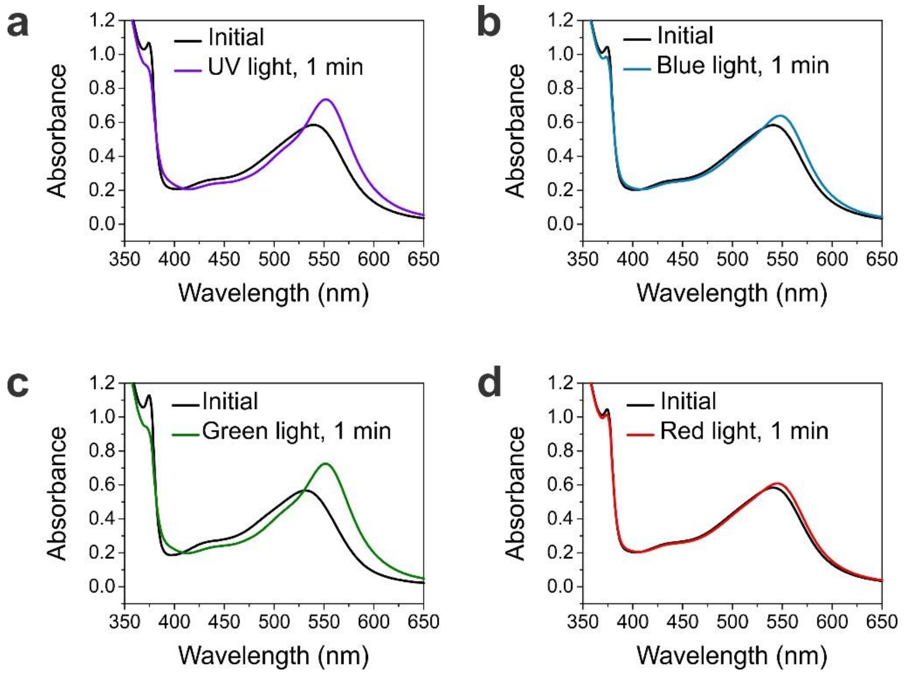

Supplementary Figure 9. Photosubstitution of the Ru-MTE complex induced by light with different wavelengths in water. UV-vis absorption spectra of Ru-MTE complex under **a** UV light (365 nm, 50 mW cm^{-2}, 1 min), **b** blue light (470 nm, 50 mW cm^{-2}, 1 min), **c** green light (530 nm, 50 mW cm^{-2}, 1 min), and **d** red light (656 nm, 50 mW cm^{-2}, 1 min) irradiation. [Ru] = 1 mM, [MTE] = 10 mM.

Supplementary Note 1. The photoresponsiveness of the Ru-thioether complex in water

We studied the photoresponsiveness of the Ru-thioether complex in water (Supplementary Figure 9). First, the aqueous solution of the Ru complex and MTE was kept in the dark to reach the equilibrium. Then, the mixture was irradiated by light with different wavelengths for 1 min. The UV-vis absorption spectra showed that all light (365 nm, 470 nm, 530 nm, and 656 nm) can trigger the photosubstitution reaction. The results are consistent with the photoresponsiveness of Ru-MTE-modified surface (Supplementary Figure 18).

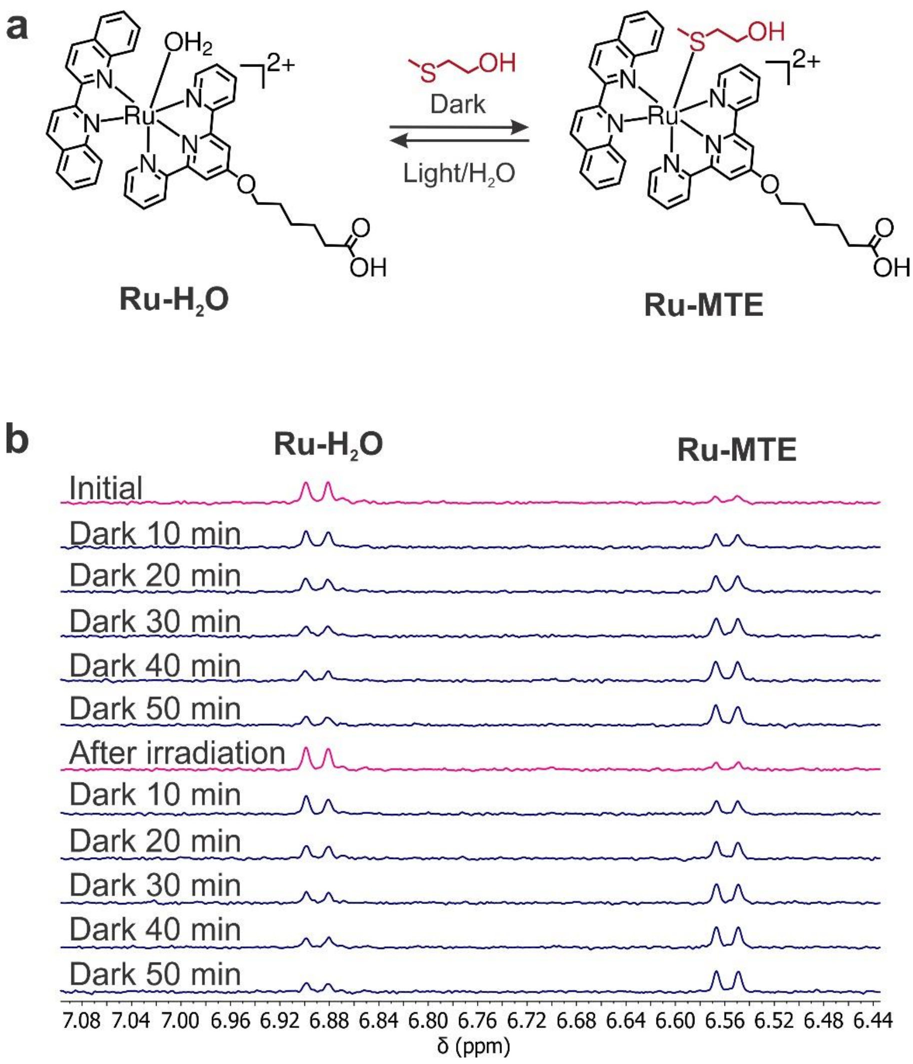

Supplementary Figure 10. Investigation of the reversible ligand substitution reaction. a Reversible process of dynamic Ru-thioether coordination chemistry. **b** ^{1}H NMR spectra (500 MHz) of the equilibrium between Ru-H_2O and Ru-MTE; after the first irradiation (the initial state) with green light and after 10, 20, 30, 40, and 50 min in the dark; after subsequent irradiation with green light and after 10, 20, 30, 40, and 50 min in the dark.

Supplementary Note 2. The equilibrium between Ru-H_2O and Ru-MTE.

We studied the equilibrium between Ru-H_2O and Ru-MTE using ^{1}H NMR spectroscopy to investigate the Ru-thioether dynamic bond (Supplementary Figure 10). A sample was prepared by adding [Ru(tpy-COOH)(biq)(Cl)]Cl (15 mg, 0.019 mmol) and $AgPF_6$ (11mg, 0.045 mmol) into a MeOD (500 µL)/D_2O (150 µL) mixture, and then added MTE (25 mg, 0.27 mmol) into the mixture. The qualitative ^{1}H NMR spectra were taken at 298K with 32 scans. In the initial state, there are two B1 doublets at 6.89 and 6.56 ppm that correspond to the signals from Ru-H_2O and Ru-MTE, respectively. To make sure the cleavage of Ru-MTE in the initial state, we measured the sample after the first irradiation with green light (530 nm, 40 mW cm^{-2}) for 60 min. However, there was still a signal of the Ru-MTE because there was a time delay between the end of the irradiation and the first measurement because the irradiation with light was done outside of the NMR spectrometer. This time delay allowed MTE to coordinate with the Ru center. In the dark, the relative intensity of the doublets of Ru-MTE at 6.56 ppm increased with time, while the doublet of Ru-H_2O at 6.89 ppm decreased. This result showed that the Ru-thioether bond spontaneously formed in the dark. After 50 min in the dark, the reaction reached a steady state. The doublet of Ru-H_2O at 6.89 ppm did not disappear because of an existing equilibrium. Notably, two factors affect the coordination rate, i.e., the temperature and concentration of thioether. However, the coordination still reaches an equilibrium even at a higher temperature or concentration[2,3]. To study the reversible process, the NMR tube was irradiated with green light (530 nm, 40 mW cm^{-2}) for 60 min again. After light irradiation, the relative intensities of the doublets of Ru-H_2O and Ru-MTE returned to their original states, which indicated that the Ru-thioether bond was cleaved by green light. After 50 min in the dark, the Ru-thioether bond was successfully recovered and the signal of Ru-H_2O and Ru-MTE returned to the equilibrium.

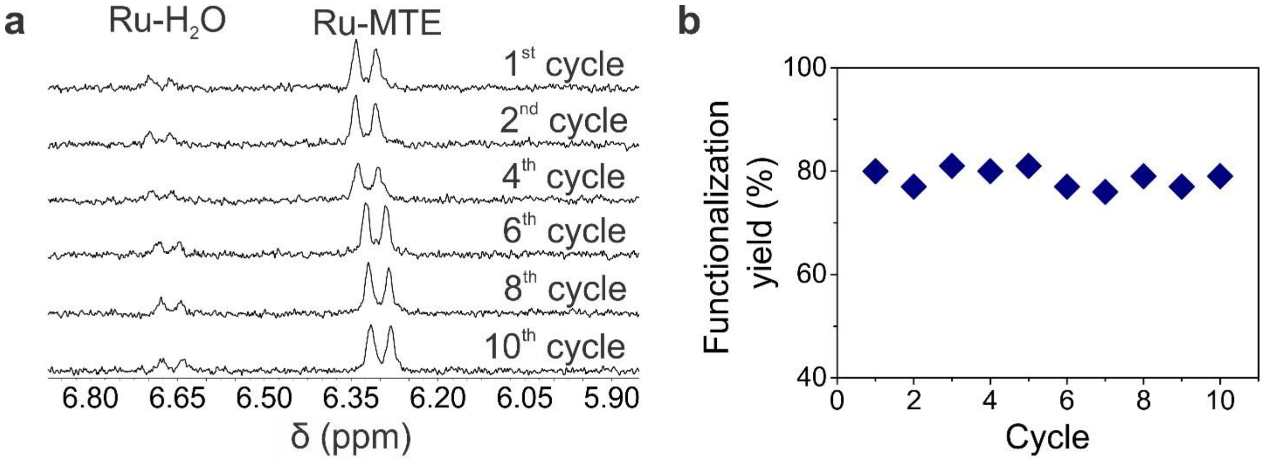

Supplementary Figure 11. Functionalization yield. a ^{1}H NMR spectra showing the reversibility of the dynamic Ru-thioether coordination chemistry for 10 cycles. **b** The functionalization yield of Ru-MTE in the dark in each cycle. The sample was measured by ^{1}H NMR during it was under light/dark treatment for 10 cycles (250 MHz, D_2O). The functionalization yield was calculated from integration of the doublets of Ru-H_2O and Ru-MTE. [Ru] = 5.3 mM, [MTE] = 53 mM.

Supplementary Note 3. Functionalization yield.

To investigate the functionalization yield, we measured the ratio of Ru-H_2O and Ru-MTE in D_2O after each irradiation/dark cycle using ^{1}H NMR (Supplementary Figure 11). The ^{1}H NMR spectra showed that the doublets of Ru-H_2O and Ru-MTE during 10 cycles. The functionalization yield of Ru-MTE was calculated from integration of the doublets of Ru-H_2O and Ru-MTE. The functionalization yield of MTE coordinated with the Ru center was approximately 80% at each cycle, which revealed that there was no significant photodamage over several cycles.

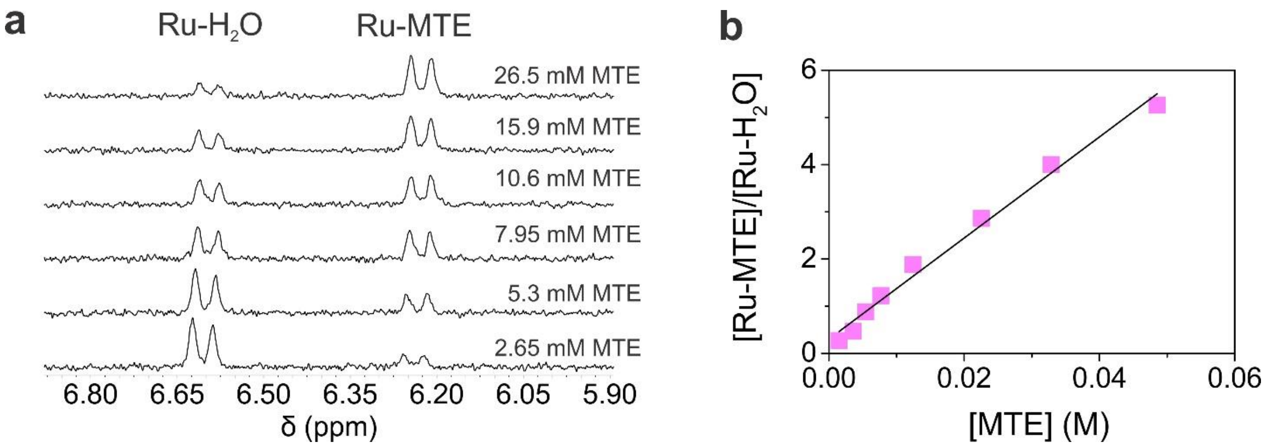

Supplementary Figure 12. Equilibrium constant. a The ^{1}H NMR spectra of the equilibrium between Ru-H_2O and Ru-MTE with different concentrations of MTE (250 MHz, D_2O, 298K). **b** Plots of the ratio [Ru-MTE]/[Ru-H_2O] vs the equilibrium concentration of free MTE at 298 K. The total concentration of Ru ([Ru]) is 5.3 mM in each sample. Equilibrium constant *K* was corresponded to the slope of the line. *K* was 107 ± 4 M^{-1}.

Supplementary Note 4. Calculation of the equilibrium constant.

The equilibrium constant *K* was determined by the following equation:

$$\frac{[Ru\text{-}MTE]}{[Ru\text{-}H_2O]} = K \cdot [MTE] \qquad (1)$$

where [Ru-H_2O] is the concentration of Ru-H_2O; [Ru-MTE] is the concentration of Ru-MTE; [MTE] is the equilibrium concentration of free MTE .

To calculate the *K*, Ru-H_2O (5.3 mM) was mixed with different amounts of MTE in D_2O, respectively. After keeping in the dark overnight, the samples were measured by ^{1}H NMR (Supplementary Figure 12a). The relative concentration ratio of Ru-H_2O and Ru-MTE was calculated by integration of the doublets of Ru-H_2O and Ru-MTE. *K* was corresponded to the slope

of the line, which was obtained from the plot of the ratio [Ru-MTE]/[Ru-H_2O] vs the equilibrium concentration of free MTE (Supplementary Figure 12b). The equilibrium constant *K* was 107 ± 4 M^{-1}.

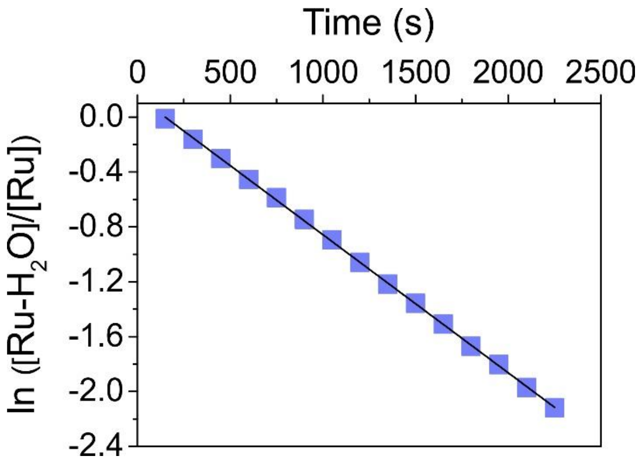

Supplementary Figure 13. Rate constant. Plot of ln ([Ru-H_2O]/ [Ru]) vs time for the Ru-thioether coordination. T = 298 K, [Ru] = 0.25 mM, [MTE] = 2.5 mM. The pseudo first-order rate constant $k_1' = 1 \times 10^{-3}$ s^{-1}.

Supplementary Note 5. Calculation of the rate constant.

The rate constant was calculated according to the following two steps[2]:

(i) Extinction coefficients. The extinction coefficients of Ru-H_2O and Ru-MTE were determined using UV-vis absorption spectroscopy. The extinction coefficient of Ru-H_2O ($\varepsilon_{\lambda}^{\text{Ru-H}_2\text{O}}$) was obtained by a traditional method. First, different concentrations of Ru-H_2O were prepared in water (2×10^{-5} M, 4×10^{-5} M, 6×10^{-5} M, 8×10^{-5} M and 1×10^{-4} M), respectively. Then, 3 mL of each sample was measured by UV-vis absorption spectroscopy. Finally, $\varepsilon_{\lambda}^{\text{Ru-H}_2\text{O}}$ was obtained from the slope of the absorption vs concentration of Ru-H_2O at each wavelength. The values of $\varepsilon_{550}^{\text{Ru-H}_2\text{O}}$ and $\varepsilon_{535}^{\text{Ru-H}_2\text{O}}$ are 9692 L mol^{-1} cm^{-1} and 8082 L mol^{-1} cm^{-1}, respectively.

The extinction coefficient of Ru-MTE was obtained by a different method due to the thermodynamic equilibrium of Ru-H_2O and Ru-MTE in water. First, four different Ru-H_2O and MTE mixture solutions were prepared. In these mixture solutions, the [Ru-H_2O] (and the total concentration of Ru [Ru]) was 4.3 × 10^{-5} M, 8.6 × 10^{-5} M, 1.3 × 10^{-4} M, and 1.7 × 10^{-4} M, respectively. [MTE] in all mixtures was 0.16 M. All mixtures were measured by UV-vis absorption spectroscopy. As [MTE] was the same, the ratio [Ru-MTE]/[Ru-H_2O] is a constant.

$$K' = \frac{[\textit{Ru-MTE}]}{[\textit{Ru-H}_2\textit{O}]} = K \cdot [\textit{MTE}] \qquad (2)$$

According to the $[\textit{Ru}] = [\textit{Ru-MTE}] + [\textit{Ru-H}_2\textit{O}]$, the Eq. (2) can be replaced by Eq. (3):

$$r = \frac{[\textit{Ru-MTE}]}{[\textit{Ru}]} = \frac{K'}{K' + 1} \qquad (3)$$

The value of r can be obtained from Eq. (3), and the value of $\varepsilon_{\lambda}^{\text{Ru-H}_2\text{O}}$ is determined above. Thus, the extinction coefficients of Ru-MTE can be calculated by Eq. (4):

$$\varepsilon_{\lambda}^{\text{Ru-MTE}} = \frac{\varepsilon_{\lambda}^{\text{Ru}} - (1 - r) \cdot \varepsilon_{\lambda}^{\text{Ru-H}_2\text{O}}}{r} \qquad (4)$$

The values of $\varepsilon_{550}^{\text{Ru-MTE}}$ and $\varepsilon_{535}^{\text{Ru-MTE}}$ are 5529 L mol^{-1} $cm^{-1}$and 6790 L mol^{-1} cm^{-1}, respectively.

(ii) Rate constant. The rate constant was also characterized using UV-vis absorption spectroscopy. First, the concentrations of Ru-H_2O and Ru-MTE in the reaction were calculated using a two-wavelength method. As the reaction is thermodynamic, the absorption at two different wavelengths can be expressed as:

$$A_{\lambda 1} = \varepsilon_{\lambda 1}^{\text{Ru-H}_2\text{O}} \cdot l \cdot [Ru\text{-}H_2O] + \varepsilon_{\lambda 1}^{\text{Ru-MTE}} \cdot l \cdot [Ru\text{-}MTE] \quad (5a)$$

$$A_{\lambda 2} = \varepsilon_{\lambda 2}^{\text{Ru-H}_2\text{O}} \cdot l \cdot [Ru\text{-}H_2O] + \varepsilon_{\lambda 2}^{\text{Ru-MTE}} \cdot l \cdot [Ru\text{-}MTE] \quad (5b)$$

According to Eq. (5b), [Ru-H_2O] can be expressed to Eq. (6):

$$[Ru\text{-}H_2O] = \frac{A_{\lambda 2} - \varepsilon_{\lambda 2}^{\text{Ru-MTE}} \cdot l \cdot [Ru\text{-}MTE]}{\varepsilon_{\lambda 2}^{\text{Ru-H}_2\text{O}} \cdot l} \quad (6)$$

Thus, after introducing Eq. (6) to Eq. (5a), the [Ru-MTE] can be expressed as (l = 1 cm):

$$[Ru\text{-}MTE] = \frac{A_{\lambda 1} \cdot \varepsilon_{\lambda 2}^{\text{Ru-H}_2\text{O}} - A_{\lambda 2} \cdot \varepsilon_{\lambda 1}^{Ru-H2O}}{\varepsilon_{\lambda 2}^{\text{Ru-H}_2\text{O}} \cdot \varepsilon_{\lambda 1}^{\text{Ru-MTE}} - \varepsilon_{\lambda 1}^{\text{Ru-H}_2\text{O}} \cdot \varepsilon_{\lambda 2}^{\text{Ru-MTE}}} \quad (7)$$

The rate law of Ru-thioether coordination reaction in water can be expressed as:

$$\frac{d[Ru\text{-}MTE]}{dt} = -\frac{d[Ru\text{-}H_2O]}{dt} = k_1 \cdot [MTE] \cdot [Ru\text{-}H_2O] - k_{-1} \cdot [Ru\text{-}MTE] \quad (8)$$

where, k_1 is the second-order rate constant. As the [MTE] is in large excessed, the pseudo first-order rate constant $k_1^{'}$ can be defined as $k_1^{'} = k_1 \cdot$ [MTE]. Thus, the Eq. (8) can be replace by:

$$\frac{d[Ru\text{-}MTE]}{dt} = -\frac{d[Ru\text{-}H_2O]}{dt} = k_1^{'} \cdot [Ru\text{-}H_2O] - k_{-1} \cdot [Ru\text{-}MTE] \quad (9)$$

The Eq. (9) can simplified to Eq. (10). Thus, the pseudo first-order rate constant $k_1^{'}$ is defined by Eq. (10):

$$\frac{d[Ru\text{-}MTE]}{dt} = -\frac{d[Ru\text{-}H_2O]}{dt} = k_1^{'} \cdot [Ru] - (k_{-1} + k_1^{'}) \cdot [Ru\text{-}MTE] \quad (10)$$

The thermal back coordination (k_{-1}) is negligible and $k_1^{'}$ is obtained from the slope of the plot of ln ([Ru-H_2O]/ [Ru]) vs time (Supplementary Figure 13). The values of $k_1^{'}$ is 1×10^{-3} s^{-1}.

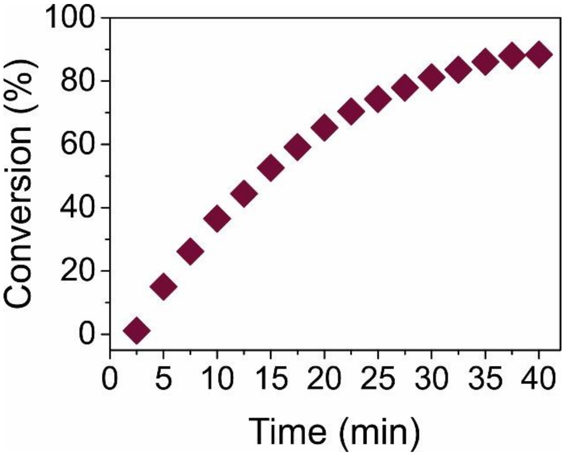

Supplementary Figure 14. Conversion of Ru-MTE in dark. The conversion obtained from UV-vis measurement. T = 298 K, [Ru] = 0.25 mM, [MTE] = 2.5 mM.

Supplementary Note 6. Calculation of conversion of Ru-MTE in dark.

We calculated the conversion of Ru-MTE according to the Eq. (11).

$$Conversion = \frac{[Ru\text{-}MTE]}{[Ru]} \cdot 100\% \tag{11}$$

The conversion of Ru-MTE at equilibrium is approximately 88% (Supplementary Figure 14), which is comparable to that measured by ^{1}H NMR (~ 80%, Supplementary Figure 11). In contrast, the efficiency of surface-bond reaction (~ 62%, Supplementary Table 1) is lower than that in solution because of the steric hindrance of the surface and less mobility of the grafted Ru complexes.

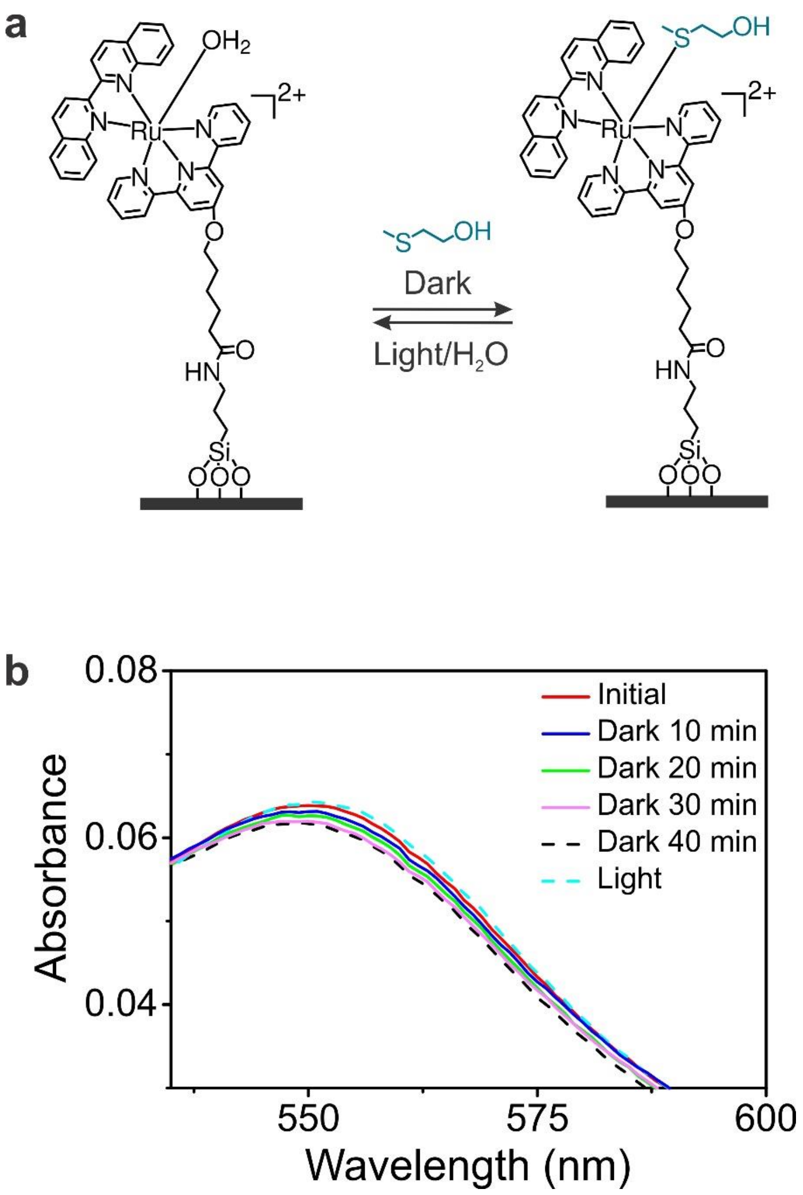

Supplementary Figure 15. Characterization of the reversible Ru-thioether coordination on surface. **a** Schematic illustration of the reversible process on surface. **b** UV-vis absorption spectra of the Ru-H_2O-modified surface just immersed in an MTE aqueous solution (10 mM) in a quartz cuvette in the dark for 10, 20, 30, and 40 min, and then irradiated with green-light irradiation (530 nm, 40 mW cm^{-2}) for 10 min. The spectral change was similar to that observed in solution, which indicated that the ligand substitution on the surface was also reversible.

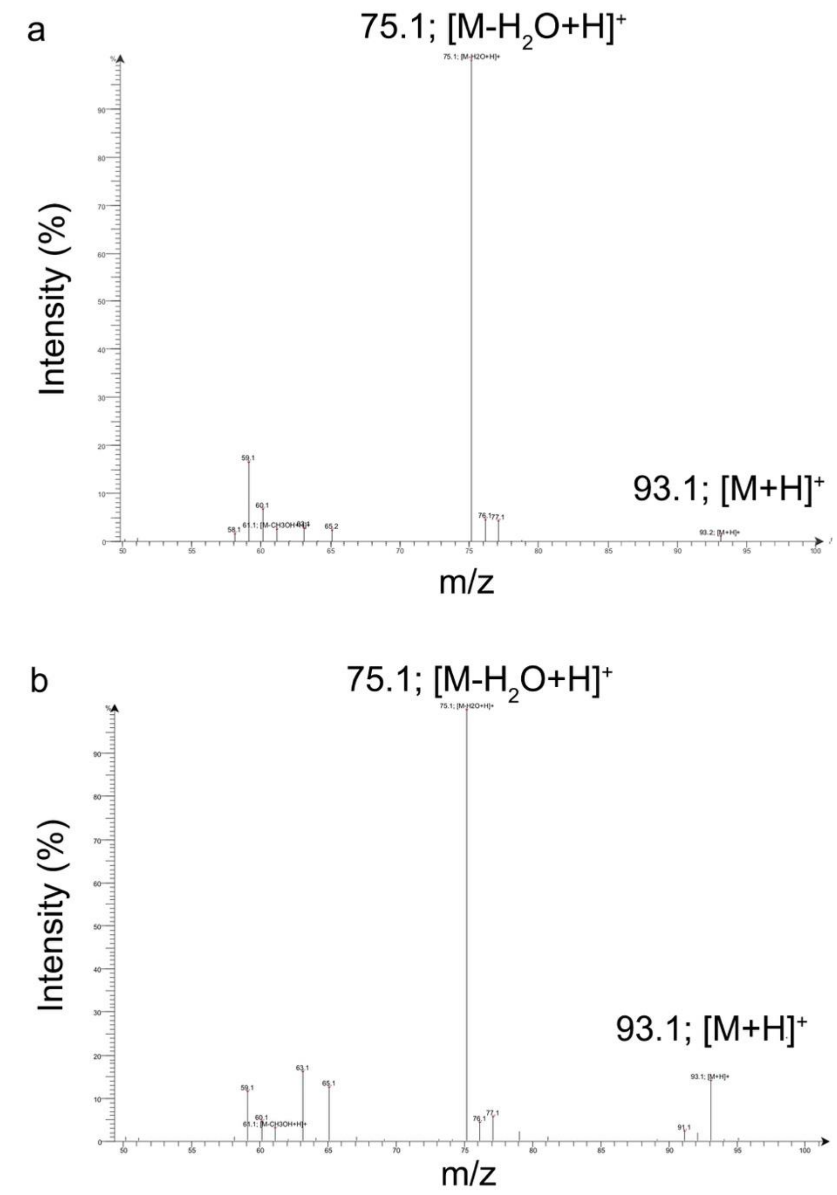

Supplementary Figure 16. Mass spectrometry analysis of the MTE released from the surface. Mass spectra of **a** the non-coordinated MTE and **b** the MTE that photo-released from the Ru-MTE-modified surface.

Supplementary Note 7. Mass spectrometry analysis of the MTE released from the surface.

The thioether MTE is intact after releasing from the surface. We immersed ten Ru-MTE-modified substrates into water and used green light (530 nm, 40 mW cm^{-2}) to irradiate the substrates for 10 min, respectively. After collecting and drying the solution, the cleaved MTE in the solution were characterized by mass spectrometry (Expression-L Compact Mass Spectrometer, Advion). The mass spectrum showed that the molecular weight of the cleaved MTE is comparable to that of the non-coordinated MTE, which shows the cleaved MTE is intact (Supplementary Figure 16).

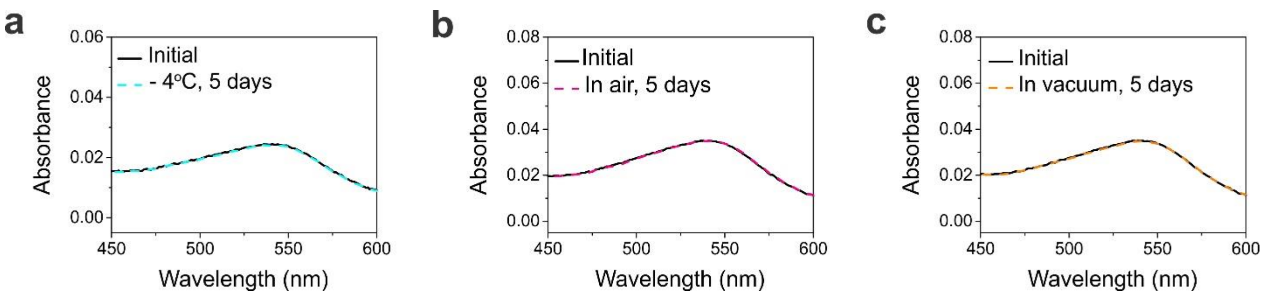

Supplementary Figure 17. The stability of the Ru-MTE-modified surface under different storage conditions in the dark. UV-vis absorption spectra of Ru-MTE-modified surfaces before and after storage in **a** fridge (- 4°C), **b** air, and **c** vacuum for 5 days.

Supplementary Note 8. The stability of the Ru-MTE-modified surface under different storage conditions in the dark.

We checked the stability of the surfaces stored in three conditions: fridge (- 4°C), air, and vacuum for 5 days in the dark. After that, the surfaces were washed with water and acetone and dried in the dark. The UV-vis absorption spectra showed that no band shift for all conditions, which indicates that the surface was stable under these conditions for 5 days (Supplementary Figure 17).

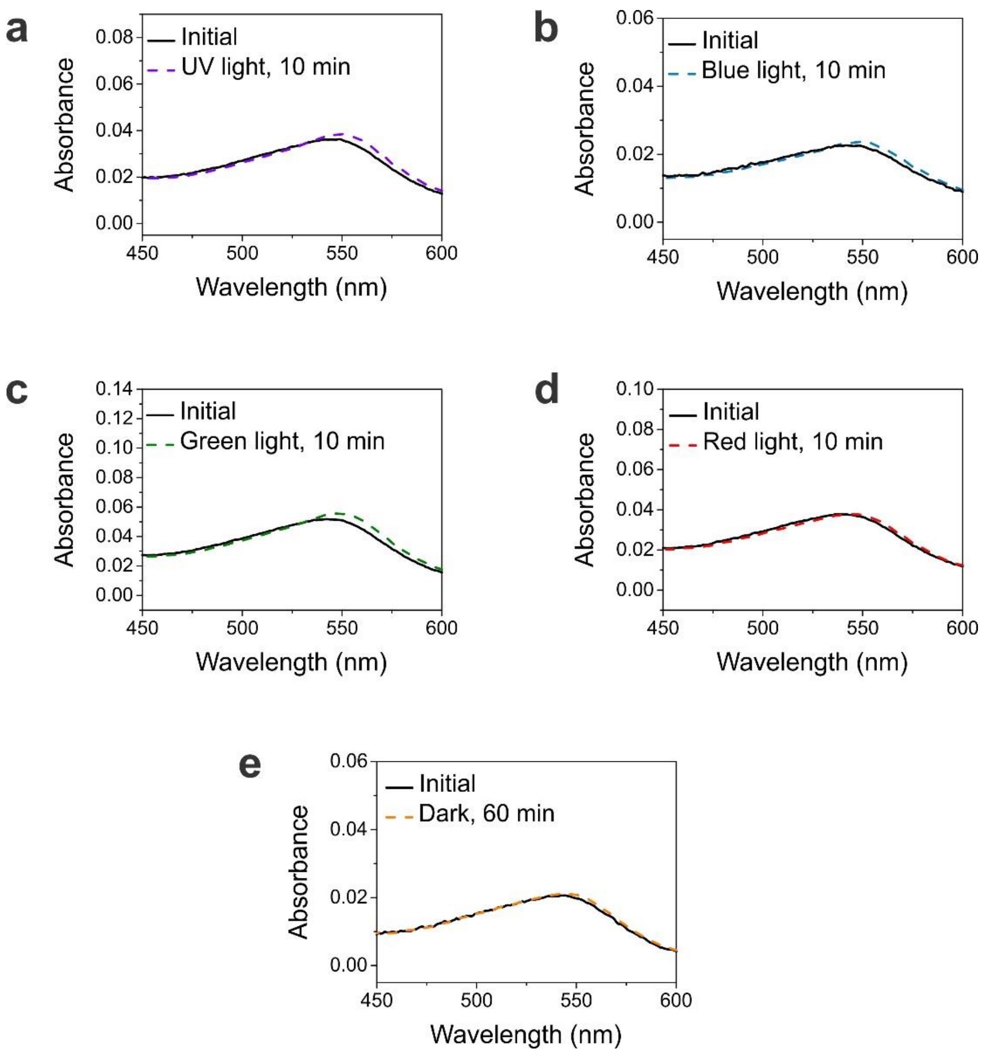

Supplementary Figure 18. The stability of the Ru-MTE-modified surfaces under light irradiation or in the dark in water. UV-vis absorption spectra of Ru-MTE-modified surfaces under **a** UV light (365 nm, 40 mW cm^{-2}, 10 min), **b** blue light (470 nm, 40 mW cm^{-2}, 10 min), **c** green light (530 nm, 40 mW cm^{-2}, 10 min), **d** red light (656 nm, 40 mW cm^{-2}, 10 min) irradiation, and **e** in the dark (60 min).

Supplementary Note 9. The stability of the Ru-MTE-modified surfaces under light irradiation or in the dark in water.

To investigate the stability of the surface, we used UV (365 nm), blue (470 nm), green (530 nm) and red (656 nm) light to irradiate the surface immersed in water and in air, respectively. First, the Ru-MTE-modified surfaces were irradiated in water with light. After washing with water and acetone and drying in the dark, the stability of the surfaces was evaluated using UV-vis absorption spectroscopy. The UV-vis spectra showed all absorption bands were shifted (Supplementary Figure 18a-d), which means the MTE were cleaved from the surfaces after light irradiation in water. The shift of the absorption band under green light is bigger than that under blue light and red light. This is because the absorption maximum of Ru-MTE is at 535 nm. There was a very minor change after the surface was kept in water in the dark for 60 min (Supplementary Figure 18e). The surface is photosensitive in water.

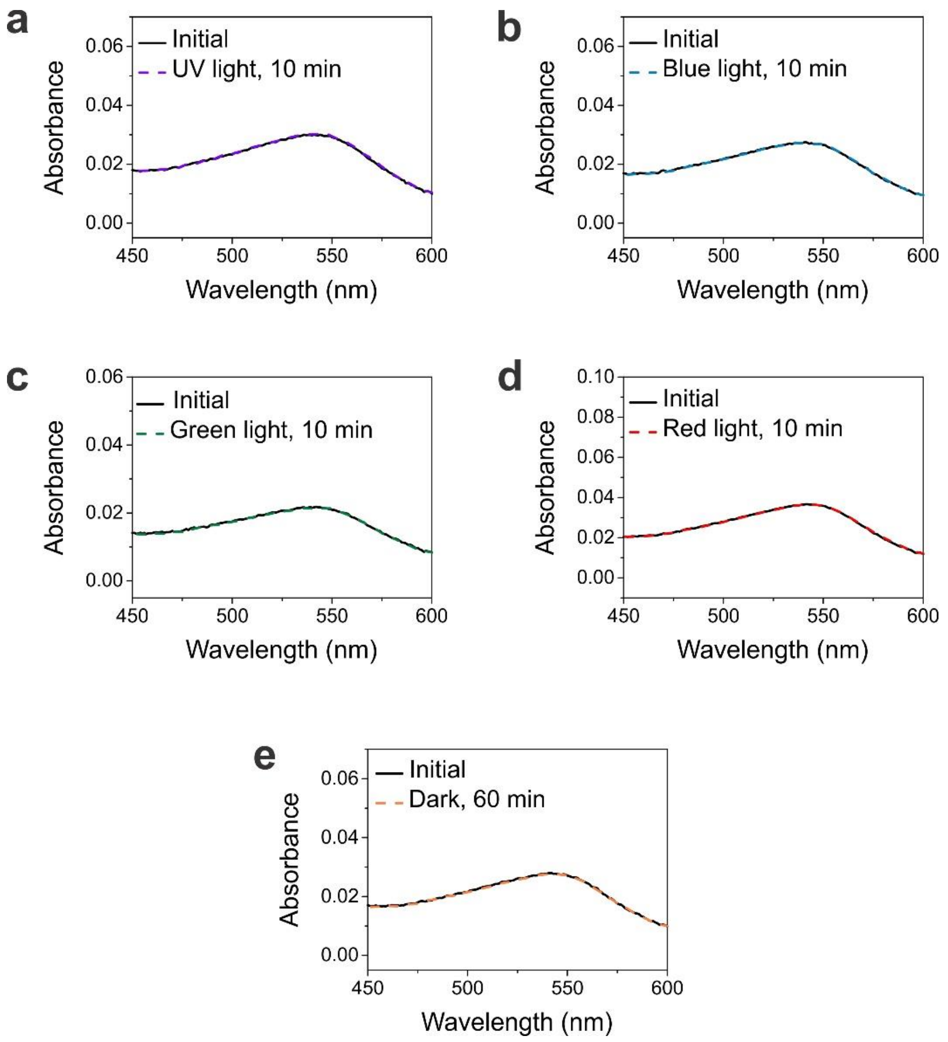

Supplementary Figure 19. The stability of the Ru-MTE-modified surfaces under light irradiation or in the dark in air. UV-vis absorption spectra of Ru-MTE-modified surfaces under **a** UV light (365 nm, 40 mW cm^{-2}, 10 min), **b** blue light (470 nm, 40 mW cm^{-2}, 10 min), **c** green light (530 nm, 40 mW cm^{-2}, 10 min), **d** red light (656 nm, 40 mW cm^{-2}, 10 min) irradiation, and **e** in the dark (60 min).

Supplementary Note 10. The stability of the Ru-MTE-modified surfaces under light irradiation or in the dark in air.

The surface is stable under light irradiation or in the dark in air. We used light with different wavelengths to irradiate the Ru-MTE-modified surfaces in air. Then, the samples were washed and dried in the dark. UV-vis absorption spectra showed that the absorption bands did not change in all groups, revealing that the Ru-MTE-modified surfaces are stable in air (Supplementary Figure 19).

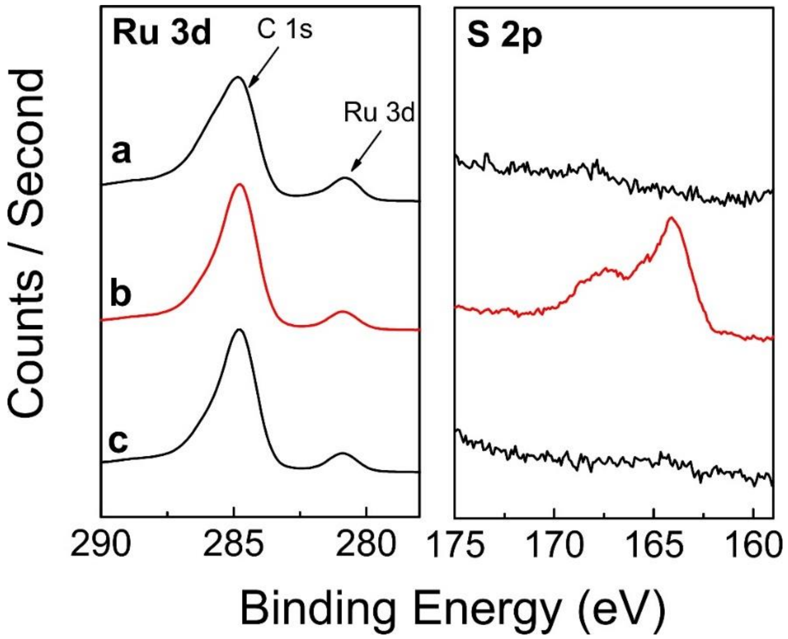

Supplementary Figure 20. XPS spectra of the different surfaces. **a** before addition of the thioether ligand MTE (Ru-H_2O-modified surface), **b** after addition of MTE in the dark (Ru-MTE-modified surface), and **c** after light irradiation and washing.

Supplementary Note 11. XPS analysis of the different surfaces.

The XPS spectra revealed that the surface is successfully modified by Ru-H_2O (Supplementary Figure 20a). The peak of S 2p appeared after MTE was grafted on the surface (Supplementary Figure 20b). After light irradiation and washing, the peak of S 2p disappeared (Supplementary Figure 20c). XPS spectra showed that MTE successfully coordinated with the Ru centers on the substrate in the dark and were released from the substrate after light irradiation.

The quantitative XPS analysis showed the percentage of the coordinated Ru centers (average 62%) is lower than that in solution (~ 80%) probably because the surface has steric hindrance effect and the grafted Ru complexes have less mobility (Supplementary Table 1).

XPS was conducted using a Kratos Axis UltraDLD spectrometer (Kratos, Manchester, England) using an Al Kα excitation source with a photon energy of 1487 eV. The data were acquired in the hybrid mode using a 0° take-off angle, defined as the angle between the surface normal and the axis of the analyzer lens. Detailed region XPS spectra were collected with setting analyzer pass energy at 80 eV. Neutralizer was always used during spectra collection and binding energy scales were further calibrated according to dominant C 1s emission at 284.8 eV.

Atomic composition of Ru and S were calculated according to the equation below, by calculating the emission peak areas (Intensity, *I*) after normalization with the supplied sensitivity factors (*SF*).

$$\frac{\%_{\mathrm{Ru}}}{\%_{\mathrm{S}}} = \frac{(I_{\mathrm{Ru}}/SF_{\mathrm{Ru}})}{(I_{\mathrm{S}}/SF_{\mathrm{S}})} \qquad (12)$$

Linear background was always used for peak quantification.

The surfaces were measured by XPS for 10 cycles of binding and cleavage of the thioether ligand, respectively. The atomic composition of Ru and S, and functionalization yield of MTE coordinated with the Ru center on the surface after the equilibrium in the dark at different cycles are shown in Supplementary Table 1. The functionalization yield showed no significant change after 10 cycles, which is consistent to that in solution (Supplementary Figure 11). In each cycle, the peak of S was not detected after light irradiation (the atomic composition of S was 0%), which means that the thioether ligands could be all cleaved from the surface. The results demonstrated that the Ru-S coordination on the surface is reversible.

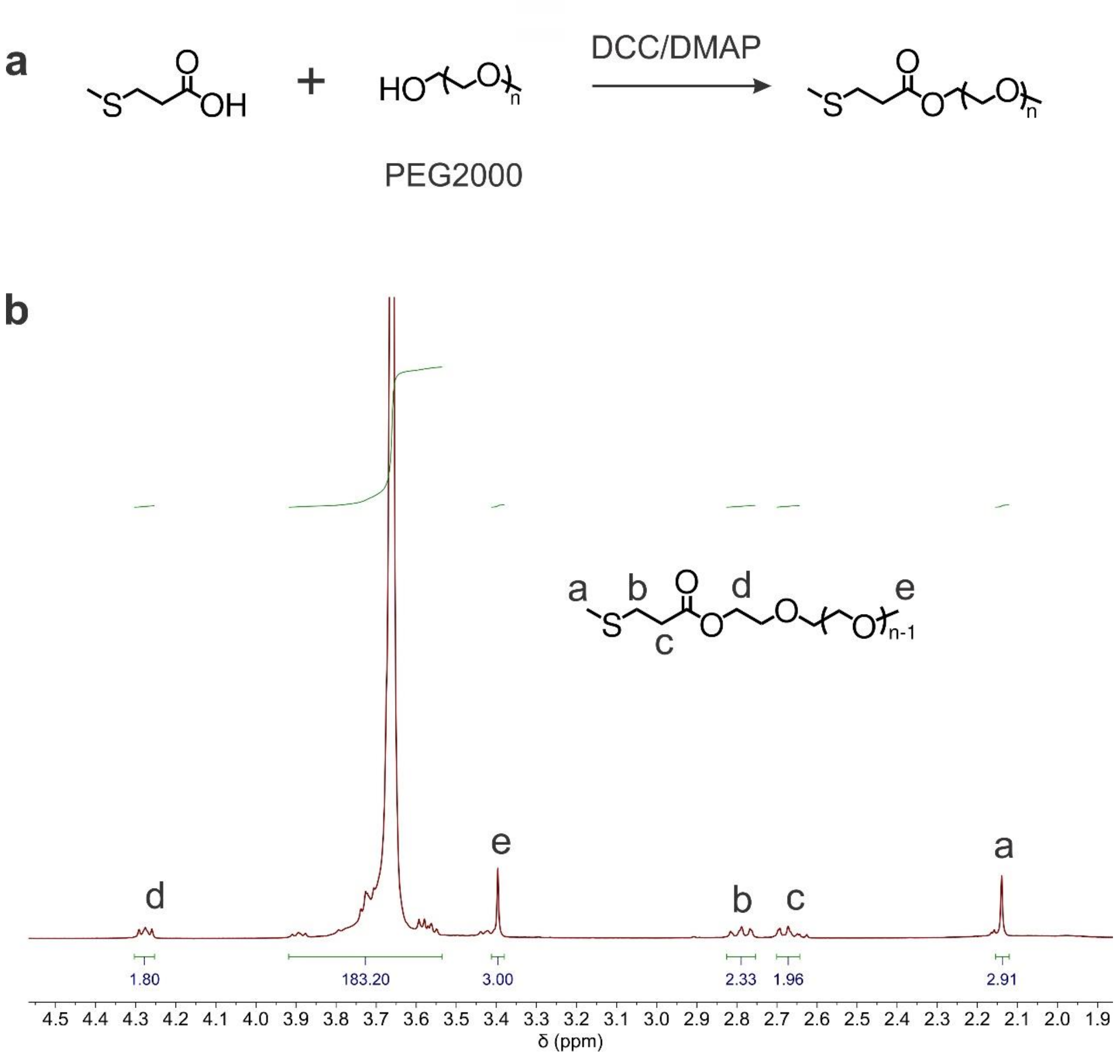

Supplementary Figure 21. Synthesis and characterization of MeSC$_2$H$_4$-PEG. a Route for synthesis of MeSC$_2$H$_4$-PEG. **b** ^{1}H NMR spectrum of MeSC$_2$H$_4$-PEG (300 MHz, CDCl$_3$).

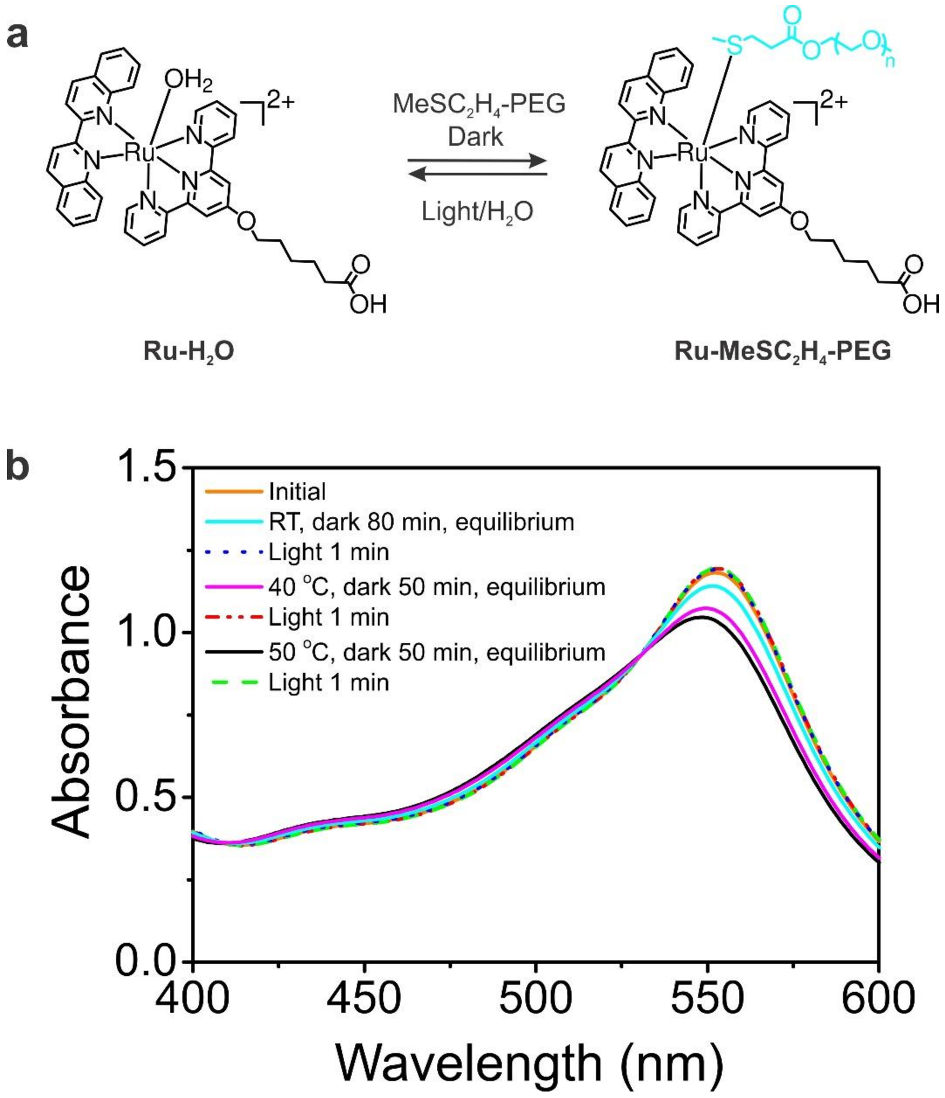

Supplementary Figure 22. Reversible coordination of $MeSC_2H_4$-PEG with the Ru complex in solution. a Schematic illustration of the reversible process. b The UV-vis absorption spectra of the H_2O/acetone mixture (7:1) containing Ru-H_2O (0.25 mM) and $MeSC_2H_4$-PEG (1.5 mM) at different temperatures in the dark at equilibrium and after green-light irradiation (530 nm, 50 mW cm^{-2}) for 1 min. The spectral change demonstrated that the ligand substitution in solution was reversible at different temperatures. The shift in the absorption band was small at room temperature (RT), which indicated the coordination efficiency was low. Importantly, the efficiency increased at elevated temperatures.

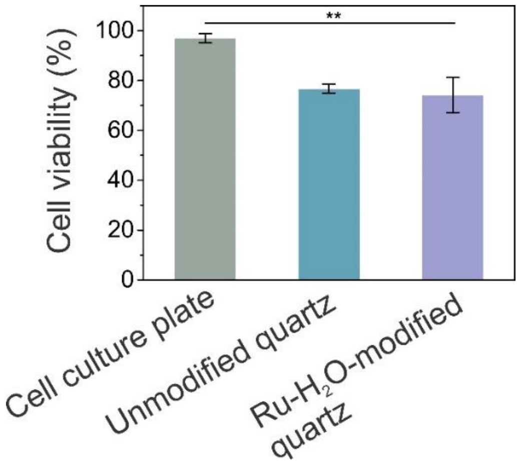

Supplementary Figure 23. The toxicity of the Ru-H_2O-modified surface. HeLa cell viability on cell culture plate (negative control), unmodified quartz surface and Ru-H_2O-modified quartz surface. ** $P < 0.001$

Supplementary Note 12. The toxicity of the Ru-H_2O-modified surface.

To evaluate the toxicity of the Ru-H_2O-modified surface, HeLa cells (1×10^5 cells well^{-1}) were seeded to the respective wells in a 24-well plate with an unmodified quartz surface and Ru-H_2O-modified quartz surface. The cells incubated for 24 h at 37 °C to attach to the surface of the samples. After the incubation, the medium was changed and the cells were incubated for 24 h. After the second incubation, the cells were detached and washed by centrifugation, resuspended in 1 mL of PBS buffer and the viability was measured using Flow Cytometry (Thermo Fisher, USA) through propidium iodide staining. The results showed that the cell viabilities in both groups were approximately 75% compared to the cell culture plate (Supplementary Figure 23). The biocompatibility of the Ru-H_2O-moidifed quartz is as good as unmodified quartz. This indicates a monolayer of the Ru complex on the surface does not strongly affect the cell viability. In addition, the Ru complex is fixed on the surface via covalent bonding. Therefore, it cannot be taken up by the cells and thus minimizes side effects.

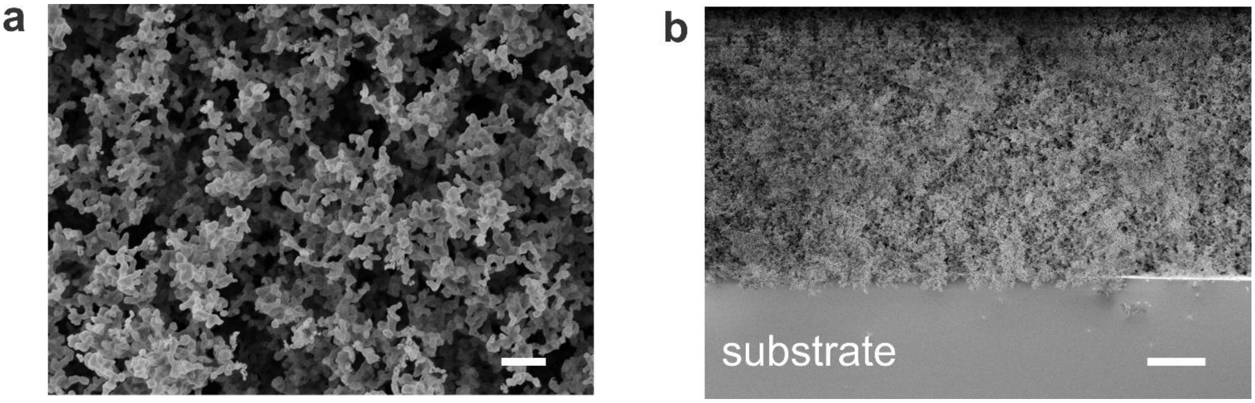

Supplementary Figure 24. The morphologies of the Ru-H_2O-modified silica coating. SEM images of **a** porous silica coating and **b** cross-section of Ru-H_2O-modified silica coating. Scale bar is 500 nm. The coating kept its nanostructure after surface modification. Scale bar is 5 μm.

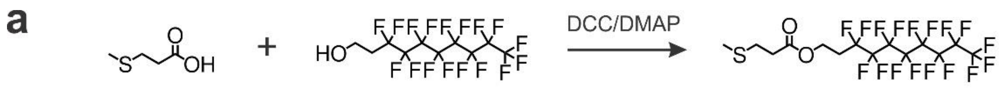

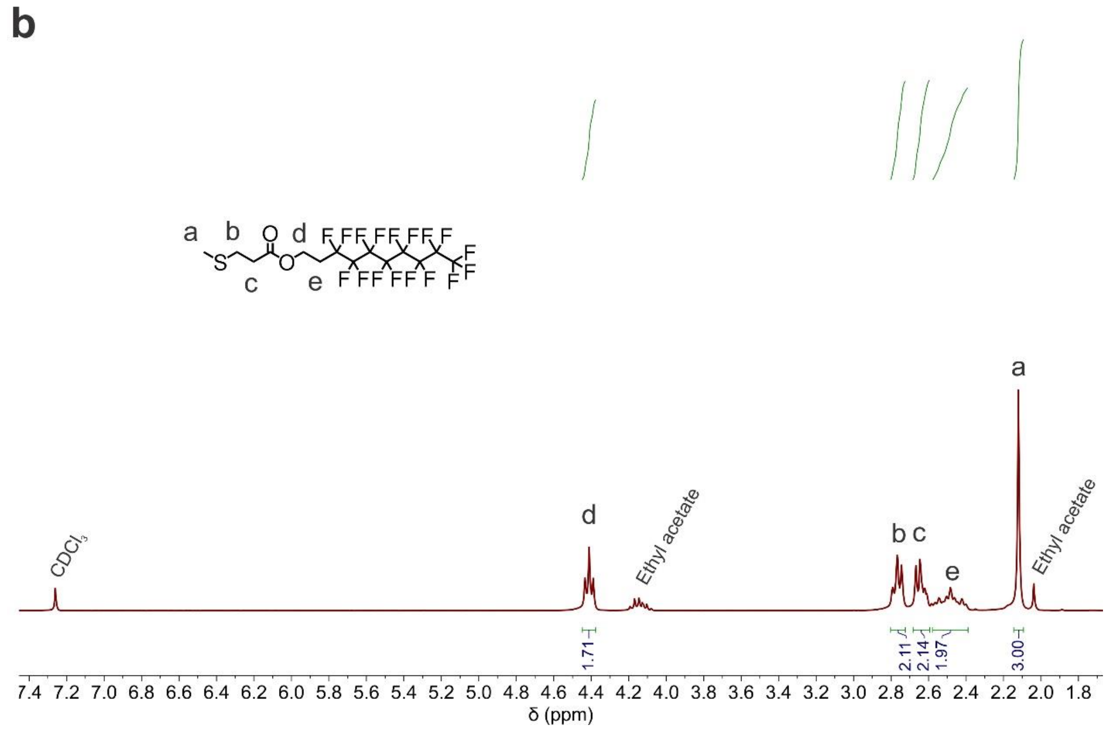

Supplementary Figure 25. Synthesis and characterization of HFDMS. a Route for synthesis of HFDMS. **b** ^{1}H NMR spectrum of HFDMS (300 MHz, $CDCl_3$).

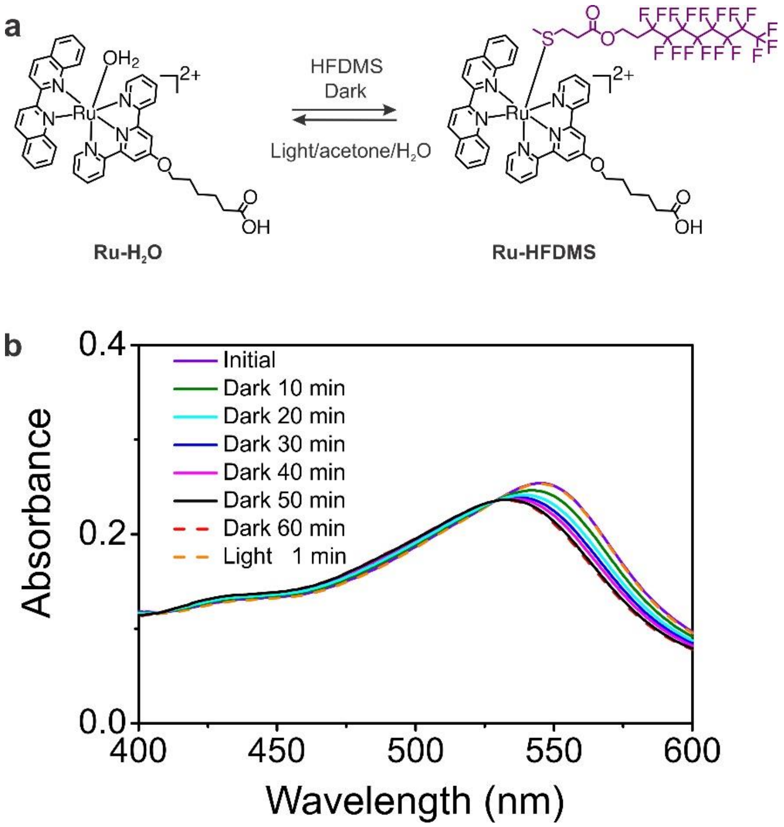

Supplementary Figure 26. Characterization of coordination of HFDMS and the Ru complex in solution. a Schematic illustration of the reversible process. **b** The UV-vis absorption spectra of acetone/H_2O (1:1) mixture containing Ru-H_2O (0.1 mM) and HFDMS (1 mM) in the dark for 10, 20, 30, 40, 50, and 60 min at 40 °C, and then irradiated by green light (530 nm, 50 mW cm^{-2}) for 1 min. The spectral change showed that the ligand substitution in solution was reversible.

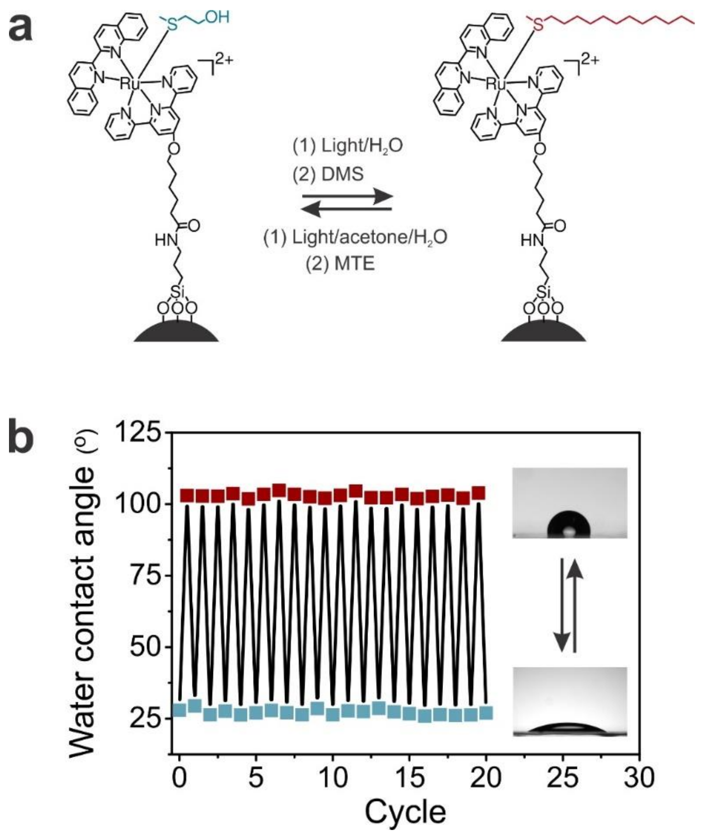

Supplementary Figure 27. Reversible hydrophilic-hydrophobic transitions. a Schematic illustration of the reversible wettability change. **b** Change of the static water contact angles when the ligands on the surface were interconverted between MTE and DMS. Blue square, MTE ligands on the surface. Dark red square, DMS ligands on the surface.

Supplementary Note 13. Reversible hydrophilic-hydrophobic transitions.

We used another thioether ligand, dodecyl methyl sulfide (DMS), to replace HFDMS (Supplementary Figure 27a). The porous silica coating was switchable between a hydrophilic state (water contact angle of 27 ± 2°) and a hydrophobic state (water contact angle of 105 ± 3°) (Supplementary Figure 27b). These results indicate that the functions of Ru-H_2O-modified surfaces can be reconfigured on demand.

Supplementary Table 1. The quantitative XPS analysis. The atomic composition of Ru and S, and functionalization yield on the surface after each irradiation/dark cycle. The compositions were determined by XPS.

Cycle	Atomic composition (%)		Functionalization yield (%)
	Ru	S	
1	61.9±1.5	38.1±1.5	61.7±3.9
2	61.3±1.9	38.7±1.9	63.2±5.2
3	66.9±0.3	33.1±0.3	49.6±0.6
4	59.7±1.1	40.3±1.1	67.7±3.1
5	64.6±1.9	35.3±1.9	54.7±4.8
6	61.7±0.9	38.3±0.9	62.1±2.2
7	58.5±1.0	41.5±1.0	70.9±2.9
8	59.9±0.1	40.1±0.1	66.9±0.4
9	59.9±0.4	40.1±0.4	66.9±1.1
10	60.8±0.5	39.2±0.5	64.4±1.3

Supplementary References

1 Andres, P. R. *et al.* New 4′-functionalized 2,2′:6′,2′′-terpyridines for applications in macromolecular chemistry and nanoscience. *Eur. J. Org. Chem.* **2003**, 3769-3776 (2003).

2 Bahreman, A., Limburg, B., Siegler, M. A., Bouwman, E. & Bonnet, S. Spontaneous formation in the dark, and visible light-induced cleavage, of a ru-s bond in water: A thermodynamic and kinetic study. *Inorg. Chem.* **52**, 9456-9469 (2013).

3 Bahreman, A. *et al.* Ruthenium polypyridyl complexes hopping at anionic lipid bilayers through a supramolecular bond sensitive to visible light. *Chem. - Eur. J.* **18**, 10271-10280 (2012).
